# Event- and Time-Driven Techniques Using Parallel CPU-GPU Co-processing for Spiking Neural Networks

**DOI:** 10.3389/fninf.2017.00007

**Published:** 2017-02-07

**Authors:** Francisco Naveros, Jesus A. Garrido, Richard R. Carrillo, Eduardo Ros, Niceto R. Luque

**Affiliations:** ^1^Department of Computer Architecture and Technology, Research Centre for Information and Communication Technologies, University of Granada Granada, Spain; ^2^Vision Institute, Aging in Vision and Action Lab Paris, France; ^3^CNRS, INSERM, Pierre and Marie Curie University Paris, France

**Keywords:** event- and time-driven techniques, CPU, GPU, look-up table, spiking neural models, bi-fixed-step integration methods

## Abstract

Modeling and simulating the neural structures which make up our central neural system is instrumental for deciphering the computational neural cues beneath. Higher levels of biological plausibility usually impose higher levels of complexity in mathematical modeling, from neural to behavioral levels. This paper focuses on overcoming the simulation problems (accuracy and performance) derived from using higher levels of mathematical complexity at a neural level. This study proposes different techniques for simulating neural models that hold incremental levels of mathematical complexity: leaky integrate-and-fire (LIF), adaptive exponential integrate-and-fire (AdEx), and Hodgkin-Huxley (HH) neural models (ranged from low to high neural complexity). The studied techniques are classified into two main families depending on how the neural-model dynamic evaluation is computed: the event-driven or the time-driven families. Whilst event-driven techniques pre-compile and store the neural dynamics within look-up tables, time-driven techniques compute the neural dynamics iteratively during the simulation time. We propose two modifications for the event-driven family: a look-up table recombination to better cope with the incremental neural complexity together with a better handling of the synchronous input activity. Regarding the time-driven family, we propose a modification in computing the neural dynamics: the bi-fixed-step integration method. This method automatically adjusts the simulation step size to better cope with the stiffness of the neural model dynamics running in CPU platforms. One version of this method is also implemented for hybrid CPU-GPU platforms. Finally, we analyze how the performance and accuracy of these modifications evolve with increasing levels of neural complexity. We also demonstrate how the proposed modifications which constitute the main contribution of this study systematically outperform the traditional event- and time-driven techniques under increasing levels of neural complexity.

## Introduction

Artificial neural networks (NNs) have been studied since the early 1940's (Mcculloch and Pitts, 1943). These NNs were born as mathematically tractable algorithms that attempted to abstract the learning mechanisms underlying our brain. The natural evolution of these NNs has lately resulted in diverse paradigms including Spiking Neural Networks (SNNs) (Ghosh-Dastidar and Adeli, 2009). These SNNs render a higher biological plausibility by bringing the concept of spike-timing into play. The idea behind the spike-timing concept is based on equipping the neural units (neurons) with the capability to emit spikes when their membrane potentials reach a specific dynamic range (firing regime). Leaky integrate-and-fire (LIF) models, for instance, emit spikes when their membrane potentials reach a specific firing threshold. When a spike is fired, it travels from the source neuron to the target neurons. The spike arrivals to the target neurons may increase or decrease their corresponding membrane potentials depending on their synaptic types and synaptic weights. The spike timing, that is, when a spike is either produced or received, constitutes the foundation for processing the neural information in SNNs and is fundamental to understand brain processing based on spike-timing codification.

Spiking Neural Networks (SNNs) will be considered as highly parallelizable algorithms in which each neural-processing unit (neuron) sends and receives data (spikes) from other neurons. These SNNs are mainly defined by three key factors:

- The neural model that defines each neural-processing unit (neurons).
- The neural network topology, that is, how the neural-processing units (neurons) are interconnected.
- The learning mechanisms that drive adaptation within the SNN at both neural and network level.

The parallelizable algorithmic nature of SNNs makes them perfect candidates for being implemented within a wide variety of specific hardware platforms, such as field programmable gate-array circuits (FPGAs) (Ros et al., 2006b; Agis et al., 2007), very large-scale integration circuits (VLSI) (Pelayo et al., 1997; Schemmel et al., 2010) or specific purpose clusters, such as SpiNNaker (Furber et al., 2013) which are better suited for parallel processing. However, the wide-spread availability of general-purpose computers has drifted the SNN algorithmic development effort toward using hardware architectures better suited for sequential processing (Neumann, 1958). These general-purpose hardware architectures designed for sequential processing (also for parallel processing in the case of GPUs) do require tailor-made (customized) solutions that allow highly parallelizable SNN algorithms to run efficiently.

Two main groups of techniques are traditionally used for simulating the neural units (neurons) of SNNs within general-purpose computers: event-driven and time-driven techniques (Brette et al., 2007). Whilst the first technique only computes the neural dynamics of a neuron when it is affected by a spiking-event (generation and propagation of neural activity), the second one iteratively updates the neural dynamics of all neurons in each simulation step time. Both groups have pros and cons (Brette et al., 2007) and the best choice depends on the SNN inner features. In this study, we have focused our efforts on developing tailor-made event-driven and time-driven solutions to overcome the architectural and processing computational problems derived from using a general-purpose computer for simulating SNNs. We have studied how the mathematical complexity of several neural models may affect the simulation accuracy and computational performance when different simulation techniques are used over a standard SNN configuration.

## Methods

In this section we further explain the mechanisms that allow us to study the relationship amongst the neural dynamic complexity, simulation accuracy, and computational performance in SNNs. The benchmark analysis of well-established neural models helps to better understand this relationship. Three well-known neural models are chosen, based on their mathematical complexity and biological plausibility (see Appendix A for further details):

- The leaky integrate-and-fire (LIF) (Gerstner and Kistler, 2002) model. It is composed of one differential equation and two exponential decay functions for both excitatory and inhibitory conductances. It is extremely efficient in computational terms; however it cannot account for a wide range of biological properties.
- The adaptive exponential integrate-and-fire (AdEx) (Brette and Gerstner, 2005) model. It is composed of two differential equations and two exponential decay functions for both excitatory and inhibitory conductances. This model is only slightly more complex than the LIF from a computational point of view; however it can be considered more biologically plausible since it is able to reproduce a wide range of firing regimes (bursting, short-term adaptation, etc.).
- The Hodgkin-Huxley (HH) (Hodgkin and Huxley, 1952) model. It is composed of four differential equations and two exponential decay functions for both excitatory and inhibitory conductances. Its neural dynamics requires more computational resources; however, its differential equations closely match the neural processes that govern the spike generation. This biophysical model reproduces rather realistic physiological properties (considering ion channel activation and deactivation features).

To run the benchmark analysis, we use the spiking neural network simulator EDLUT (Ros et al., 2006a) as the working framework. EDLUT is an efficient open source simulator mainly oriented to real time simulations that allows the processing of specific parts of the neural network using different neural dynamic evaluation techniques. To adopt an extensively used benchmark methodology, we follow the recommendations given by Brette et al. (2007) to evaluate the performance of the neural network simulation techniques (different neural network sizes, connectivity ratios, firing rates, and neural models). By means of this benchmark, we specifically evaluate how the mathematical model complexity of neurons affects the computational performance and simulation accuracy when different simulation techniques are used. The synthetic nature of the benchmark here proposed is based on previous benchmark studies (Brette et al., 2007). This benchmark emulates those neural networks which are composed of neurons with a medium to low number of input synapses (up to 1,280 input synapses per neuron). The simulation performance results may change significantly when simulating biologically realistic experiments (e.g., cortical networks) which require a much larger number of incoming synapses (up to 10,000 synapses per neuron) and firing rates (van Albada et al., 2015).

The source code is available for readers at URL: www.ugr.es/~nluque/restringido/Event_and_Time_Driven_Techniques.rar (user: REVIEWER, password: REVIEWER). All the additional material needed for the benchmark analyses (neural network, synaptic weight, input activity and neuron model description files, as well as the scripts to compile the look-up tables) are also located at the same URL.

### Neural dynamic evaluation techniques-why and for what?

EDLUT implements two different neural dynamic evaluation techniques: (a) event-driven neural techniques based on pre-computed look-up tables for CPU platforms (Ros et al., 2006a), and (b) time-driven neural techniques for both CPU (Garrido et al., 2011) and GPU (Naveros et al., 2015) platforms. EDLUT allows the combination of both neural techniques on the same simulation.

Event-driven techniques are better suited for neural network layers with low and sparse activity whose network units (neurons) present low mathematical complexity. Pre-compiling and allocating the dynamic evolution of a neural model within look-up tables allows the updating of its neural state discontinuously, i.e., at different time intervals. Thus, the neural-state update process during simulation becomes very efficient, requiring only a few accesses to these look-up tables. This technique can be applied to a wide variety of neurons of diverse mathematical complexity. The bottleneck of this computational scheme lies in two factors: (a) the dimensionality of the look-up tables, and (b) the number of look-up table readouts (the number of input and output spikes that are to be processed). The higher the neural mathematical complexity is, the higher the number of state variables, and therefore, the higher the dimensionality and the number of look-up tables needed. Higher numbers of look-up tables involve time-consuming data queries. Larger look-up tables also involve slower look-up table readouts. The number of readouts, in turn, depends on the number of events to be processed (input propagated spikes and output internal spikes; Ros et al., 2006a).

On the other hand, the time-driven neural technique outperforms the event-driven neural technique for neural network layers that present high interconnectivity ratios, high neural activities and high levels of neural mathematical complexity. This technique takes full advantage of parallel computing resources at CPU and GPU platforms. CPU time-driven techniques perform better for small and medium-size groups of neurons with a low-medium mathematical complexity (from one neuron to several thousands of neurons, depending on the mathematical complexity), whereas GPU time-driven techniques perform better for large-size groups of neurons with high mathematical complexity (from thousands to millions of neurons; Naveros et al., 2015).

When the neural network layers present high heterogeneity, both simulation techniques should be used concurrently. One example of a heterogeneous neural network can be found in the cerebellum, where the large granular layer with low and sparse activity (Luque et al., 2011a; Garrido et al., 2013b, 2016) is combined with other smaller layers dealing with higher activity rates (i.e., large-convergence neurons, such as Purkinje cells Luque et al., 2016).

### Event-driven neuron models

The implementation of event-driven neuron models has previously been stated in Mattia and Del Giudice (2000), Delorme and Thorpe (2003), Reutimann et al. (2003), Rudolph and Destexhe (2006), and Pecevski et al. (2014). Particularly, the neural simulator EDLUT implements an event-driven neural technique based on look-up tables. See Ros et al. (2006a) for a comprehensive description on EDLUT event-driven simulation techniques. Compared to previous studies (Naveros et al., 2015), in this paper we propose two independent contributions over EDLUT event-driven simulation techniques. The first contribution increases the performance by compacting the look-up table structure and improving the look-up table indexing. The second one increases the performance by improving the processing of synchronous activity. With the integration of these two new simulation techniques with the original one we can simulate each neuron model using four different configurations for event-driven neuron models: direct (the original one), combined, synchronous and combined synchronous event-driven neuron models. Below, we summarize the properties of these two new simulation techniques.

#### Combined look-up tables for complex neuron models

EDLUT pre-compiles the solution of each neural model equation into look-up tables (a look-up table per state variable). EDLUT inherently requires up to two additional state variables. The first additional state variable stores the timing of a predicted spike-firing event, whereas the second state variable stores its ending (in some cases both variables remain equal). Each look-up table dimension is indexed by a neural state variable. EDLUT neural simulation uses look-up table data queries to update the neural state variables.

The higher the mathematical complexity the neural model is, the more state variables that are needed, and the more look-up tables that are then required. Concomitantly, the dimensionality of the look-up tables also increases with the number of coupled state variables. The dimensionality and number of look-up tables are, therefore, imposed by the neural model complexity. The only way to control the look-up table size is by adjusting the look-up table granularity (the number of coordinates per dimension). Obviously, the degree of granularity has a direct impact on the accuracy and performance of the neural simulation.

**Figure 1:**
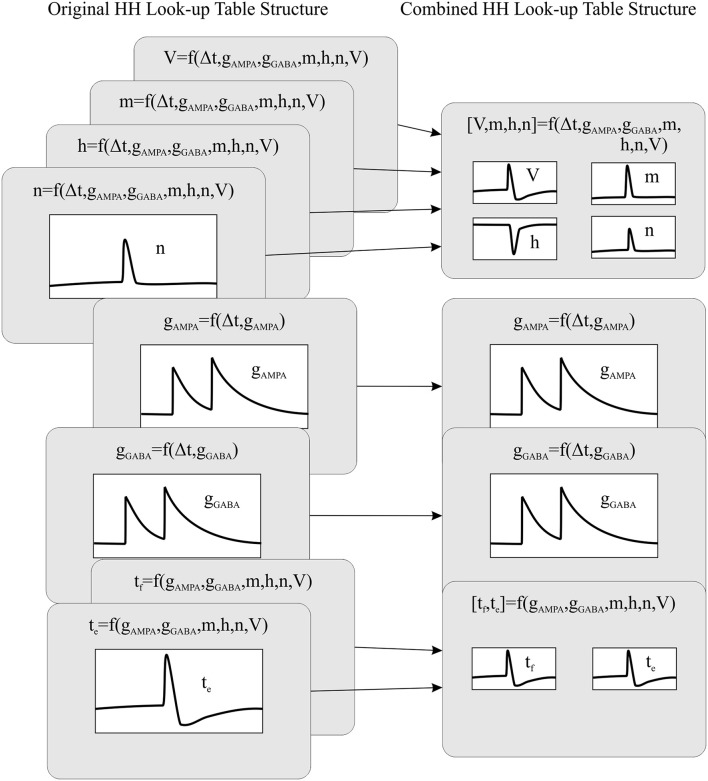
**The recombination mechanism of look-up tables for a HH model**. The left side of the panel shows the original look-up table structure (eight tables) whilst the right side of the panel shows the recombined look-up table structure (four tables).

Computing data queries using large look-up tables constitutes the most time consuming operation of all the neural dynamic evaluations. Therefore, reducing the number of look-up table readouts needed per neural model would reduce the neural dynamic evaluation time, thus increasing the overall simulation performance. Aiming to reduce the number of look-up tables, we have created a new event-driven method that recombines the look-up tables that share index values. Thus, we are able to reduce the number of look-up tables and make them more compact than the original ones (considering all of them as a whole). See Figure 1. The combined look-up tables are described as follows (see Appendix A for further details about neural model descriptions):

#### Leaky integrated-and-fire model (LIF)

- One look-up table with four dimensions storing the forecast values of the membrane potential: *V* = *f(*Δ*t, g*_*AMPA*_*, g*_*GABA*_*, V)*. The neural state variables associated to each dimension are the elapsed time since the last update (Δ*t*), the previous excitatory (*g*_*AMPA*_) and inhibitory (*g*_*GABA*_) conductances and the previous membrane potential *(V)*.
- Two look-up tables of two dimensions storing the forecast values of the excitatory and inhibitory conductances: *g*_*AMPA*_ = *f(*Δ*t, g*_*AMPA*_*)* and *g*_*GABA*_ = *f(*Δ*t, g*_*GABA*_*)*. The neural state variables associated to each dimension are the elapsed time since the last update (Δ*t*) and the previous excitatory (*g*_*AMPA*_) or inhibitory (*g*_*GABA*_) conductances.
- One look-up table of three dimensions storing the forecast values about the time of the next firing event and the ending of the refractory period *(t*_*f*_*, t*_*e*_*)* = *f(g*_*AMPA*_*, g*_*GABA*_*, V)*. The neural state variables associated to each dimension are the current excitatory (*g*_*AMPA*_) and inhibitory (*g*_*GABA*_) conductances and the current membrane potential *(V)*. Although the LIF model presents a constant refractory period (and could be easily implemented ad-hoc), we use the look-up table *t*_*e*_, which stores the evolution of the refractory period, to maintain the same event-driven simulation structure for all the neural models (LIF, AdEx and HH).

#### Adaptive exponential integrate-and-fire model (AdEx)

- One look-up table of five dimensions storing the forecast values of the membrane potential and membrane adaptation variable: *[V, w]* = *f(*Δ*t, g*_*AMPA*_*, g*_*GABA*_*, w, V)*. The neural state variables associated to each dimension are the elapsed time since the last update (Δ*t*), the previous excitatory (*g*_*AMPA*_) and inhibitory (*g*_*GABA*_) conductances, the previous membrane adaptation variable *(w)* and the previous membrane potential *(V)*.
- Two look-up tables of two dimensions storing the forecast values of the excitatory and inhibitory conductances: *g*_*AMPA*_ = *f(*Δ*t, g*_*AMPA*_*)* and *g*_*GABA*_ = *f(*Δ*t, g*_*GABA*_*)*. The neural state variables associated to each dimension are the elapsed time since the last update (Δ*t*) and the previous excitatory (*g*_*AMPA*_) or inhibitory (*g*_*GABA*_) conductances.
- One table of four dimensions for storing the forecast values about the time of the next firing event: *t*_*f*_ = *f(g*_*AMPA*_*, g*_*GABA*_*, w, V)*. The neural state variables associated to each dimension are the current excitatory (*g*_*AMPA*_) and inhibitory (*g*_*GABA*_) conductances, the current membrane adaptation variable *(w)* and the current membrane potential *(V)*. Just one additional table is needed since this model does not include a refractory period.

#### Hodgkin-huxley model (HH)

- One look-up table of seven dimensions storing the forecast values of the membrane potential and the three ionic current activation variables: *[V, m, h, n]* = *f(*Δ*t, g*_*AMPA*_*, g*_*GABA*_*, m, h, n, V)*. The neural state variables associated to each dimension are the elapsed time since the last update (Δ*t*), the previous excitatory (*g*_*AMPA*_) and inhibitory (*g*_*GABA*_) conductances, the previous ionic current activation values *(m, h, and n)* and finally the previous membrane potential *(V)*.
- Two look-up tables of two dimensions storing the forecast values of the excitatory and inhibitory conductances: *g*_*AMPA*_ = *f(*Δ*t, g*_AMPA_*)* and *g*_GABA_ = *f(*Δ*t, g*_GABA_*)*. The neural state variables associated to each dimension are the elapsed time since the last update (Δ*t*) and the previous excitatory (*g*_*AMPA*_) or inhibitory (*g*_*GABA*_) conductances.
- One look-up table of six dimensions storing the forecast values about the time of the next firing event and the start of the hyperpolarization phase: *[t*_*f*_*, t*_*e*_*]* = *f(g*_*AMPA*_*, g*_*GABA*_*, m, h, n, V)*. The neural state variables associated to each dimension are the current excitatory (*g*_*AMPA*_) and inhibitory (*g*_*GABA*_) conductances, the current ionic current activation values (*m, h*, and *n*) and finally the current membrane potential *(V)*. The look-up table *t*_e_ prevents EDLUT from duplicating internal spikes during the HH depolarization phase.

The combination of look-up tables minimizes the overall number of look-up tables for complex neuron models, since this combination allows one look-up table to store several state variables. This also means that a single look-up table readout can now update several state variables at a time. Thus, we are able to increase the computational performance of complex neuron models without modifying their accuracy.

#### Synchronous event-driven neuron models

Each time that an event-driven neuron is affected by an event, (input propagated spikes or output internal spikes) its neural state ought to be updated. After this update, EDLUT must predict whether the new neural state will make the neuron emit a spike in subsequent time steps (Ros et al., 2006a). EDLUT implements a two-stage mechanism able to handle the generation and propagation of the spikes. When EDLUT predicts a spike firing at any time, an internal-spike event is then created and inserted in the event queue. If another event modifies the spike-firing prediction, the internal-spike event is discarded; otherwise, the spike is eventually generated in the neural soma. A propagated-spike event is then generated and inserted in the event queue. This propagated-spike event is responsible for delivering the generated spike through all the neural output synapses. It holds a time stamp equivalent to the timing of its corresponding internal-spike event plus the propagation delay. When a neuron possesses several synapses with different propagation delays, the propagated-spike mechanism generates sequential propagated-spike events depending on the propagation delay values. The synaptic propagation delays are always fixed at multiples of 0.1 ms. If not, EDLUT rounds the delay within the network file to the nearest multiple of 0.1 ms.

To sum up, EDLUT triggers a three-step process in each neuron that receives a spike through a propagated-spike event (the most common event):

- When a spike arrives to an event-driven neuron, its neural state variables are updated.
- The spike increments the neural conductance associated with the synapse that propagates the spike.
- A prediction about the generation of a spike is made. If this prediction is positive, an internal spike event is inserted in the event queue.

This three-step process presents performance losses when the neural input activity is synchronous. When n spikes reach a neuron at the same time, the first n-1 predictions (and its correspondent internal spikes) would be discarded and only the *n*th taken into account. Knowing beforehand the number of synchronous spike arrivals per time step and per neuron would allow us to compute only one prediction per neuron, the *n*th prediction.

We have developed a new synchronous event-driven method able to efficiently compute synchronous neural input activity and able to generate synchronous output activity. When a group of synchronous spikes arrives to a neuron (being simulated within a synchronous event-driven method) the neural state variables are updated conjointly and a single internal spike prediction is done. This process is done in three steps:

- When the first spike of a group of synchronous spikes arrives to a synchronous event-driven neuron, its neural state variables are updated. Thus, these neural state variables will be already updated for the rest of the synchronous spikes.
- Each synchronous spike increments the neural conductance associated with the synapse that propagates the spike.
- Once EDLUT verifies that all the synchronous spikes have been processed thanks to an additional event, only one prediction about the generation of an output spike is made. If this prediction is positive, just one internal spike event is inserted in the event queue.

Thus, we only make one neural state update and one activity prediction for each group of synchronous spikes. Obviously, the additional event that helps to verify the processing of all the synchronous input spikes may cause a performance loss if the neural input activity is asynchronous (incoming activity is not grouped into tight time slots).

This new synchronous event-driven technique can also synchronize the neural spike propagation, thus allowing the efficient interconnection amongst synchronous event-driven neurons. This technique uses a parameter named synchronization period (*t*_*sync*_) that is defined in the description file of each event-driven neural model. The synchronization period value is fixed and equal or greater than zero. Each internal-spike event can be processed at any time step; however, its corresponding propagated-spike events are generated as if the internal-spike were processed at multiples of *t*_*sync*_. When *t*_*sync*_ is zero, the output activity is asynchronous and the neural network units (neurons) behave as direct event-driven neuron models. If *t*_*sync*_ is greater than zero, the output activity is then synchronous and the neural network units (neurons) can be interconnected to other synchronous event-driven models, thus increasing the overall performance but at the expense of accuracy. These synchronous models efficiently compute input activity coming from either time-driven or synchronous event-driven neuron models. Conversely, when the input activity comes from other types of event-driven neuron models the computational performance drops.

These kinds of synchronous neural layers can typically be found in neural networks that process sensory information, such as the olfactory (Schoppa, 2006) (30–70 Hz), auditory (Doesburg et al., 2012) (30–50 Hz), or visual (Eckhorn et al., 1990) (35–80 Hz) systems.

### Time-driven neuron models

EDLUT implements time-driven neuron models for both CPU (Garrido et al., 2011) and GPU (Naveros et al., 2015) platforms. These models are defined by a set of differential and non-differential equations that have to be computed during the simulation time. These equations must be solved using differential equation solvers given within a certain integration method. There are mainly two families of integration methods regarding their integration step size: a) fixed-step integration methods, and b) variable-step integration methods (Iserles, 2009).

#### Fixed-step integration methods

Fixed-step integration methods are suited for parallelization in both CPU and GPU platforms (Naveros et al., 2015) since these methods favor synchronicity during the integration process. A single integration event manages the integration process of a large number of neurons (just one event for each integration step must be generated, inserted in the event queue, extracted from the event queue, processed and deleted). Thus, the computation overhead caused by non-directly related tasks to the integration processes remains low. However, the maximum fixed-step size that can be used is constrained by the stiffness of the differential equations that define each neural model. This constraint makes fixed-step integration methods to not be well suited for solving complex neural models whose differential equations are rather stiff.

#### Variable-step integration methods

Variable-step integration methods iteratively adapt their integration step size to the neural dynamics. They are iteratively maintaining a balance between the simulation step size and the accuracy as the integration process deploys. This adaptation mechanism makes variable-step integration methods better suited for solving complex neural models whose differential equations are rather stiff. However, this flexibility comes at a cost:

- Their parallelization in CPU platforms is arduous and almost intractable in GPU platforms.
- The computation overhead caused by the estimation of the integration step size can be high.
- The integration process of each neuron has to be managed individually (one event per neuron). For each integration step an event must be generated, stored in the event queue, extracted from the event queue, processed and deleted. This overhead is determinant when the number of neurons is relatively high (thousands of neurons) and the activity is also high.
- The performance of these methods is heavily related with the neural activity. A high network activity increases the firing ratios of the neural network units (neurons). In this case, the solutions for the neural differential equations are mostly located around the neural firing working points. To maintain accuracy around a firing working point, the integration step size needs to be reduced. The computation time per neural unit is then increased.

The computational overhead caused by all these drawbacks makes these methods unadvisable for efficient simulations when the number of neural units (neurons) is relatively high. For this reason, we do not implement variable-step integration methods in EDLUT.

#### A new integration method; the Bi-fixed-step integration method

**Figure 2:**
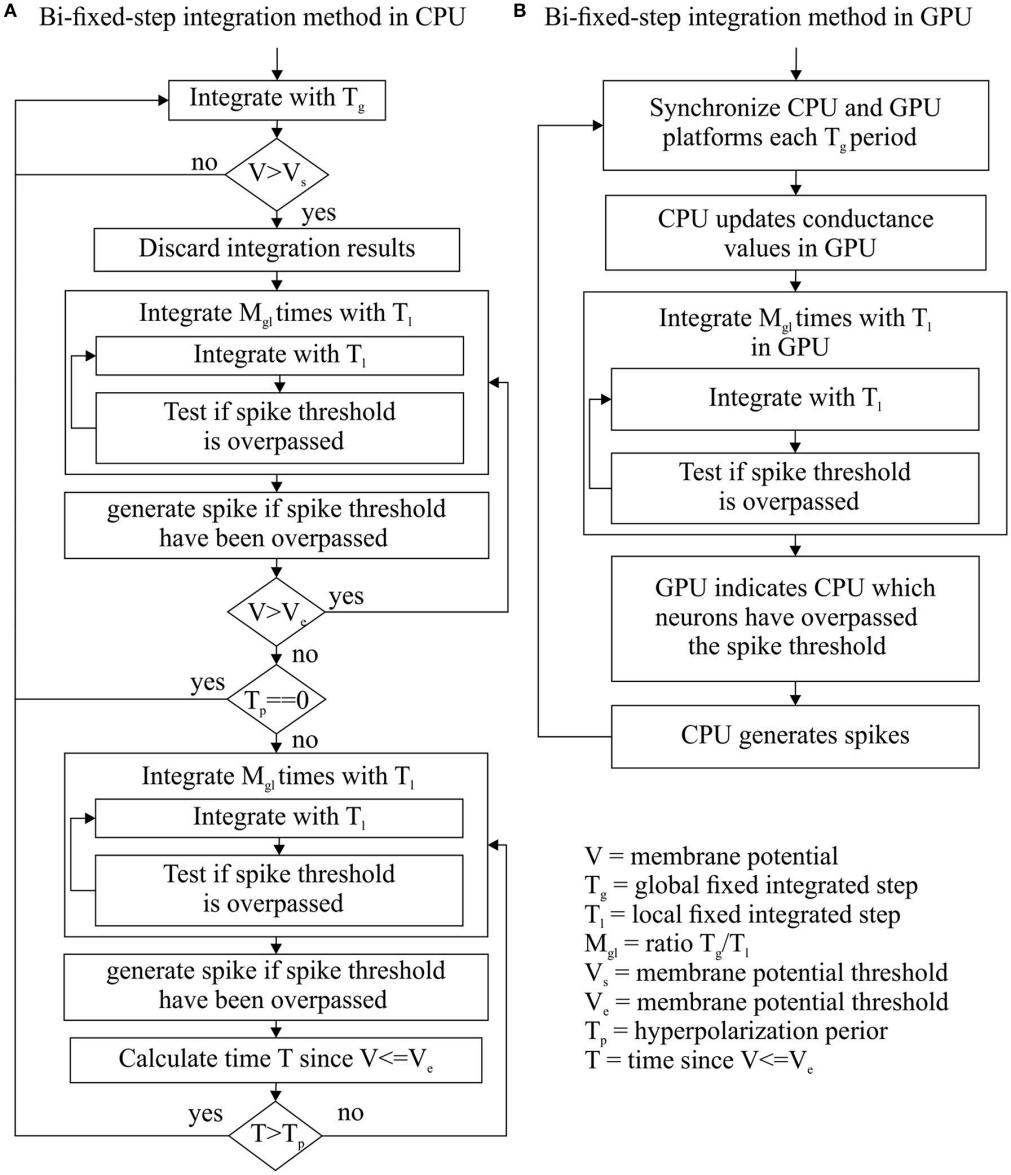
**Bi-fixed-step integration method flow diagram for CPU (A)** and GPU **(B)** platforms. Since T_l_ is divisor of T_g_, these integration methods can integrate a period of time T_g_ making M_gl_ consecutive integrations with a step-sizes of T_l_.

This new integration method tries to take advantage of the strengths and mitigate the weaknesses of fixed-step and variable-step integration methods. This integration method uses two fixed-steps for each neuron: a global fixed-step size (T_g_) and local fixed-step size (T_l_). T_g_ is multiple of T_l_ (M_gl_ = T_g_/T_l_). T_g_ synchronizes the integration processes of all network units (neurons) that are defined by the same neural model. This allows us to manage the integration process of a group of similar neurons using just a single integration event, as the fixed-step integration methods do. On the other hand, T_l_ can scale down the integration step size of one neuron when needed. When T_l_ is used instead of T_g_, the integration method performs M_gl_ consecutive integrations within T_g_. This allows us to adapt the integration step size to the dynamic evolution of each neuron, as the variable-step integration methods do. Figure 2 shows how the implementation of this integration method within CPU and GPU platforms differs in order to cope with their different hardware properties.

When EDLUT runs a simulation, the generated “events” are sorted depending on their time stamps in an event queue. When a new event is processed, its corresponding time stamp establishes the new “simulation time.” A new bi-fixed integration event produces a simulation time updating which is multiple of the global time step. The spike generation process cannot be triggered at any local time step but at the global step time, otherwise the generated spikes would carry incoherent time stamp values (lower than the actual simulation time). Therefore, the spikes to be generated are detected at local time steps but only generated at global time steps.

##### Bi-fixed-step integration method for differential equation solvers in CPU

Two additional elements per neuron model are defined: a hysteresis cycle given by two membrane potential thresholds (V_s_ as the upper bound and V_e_ as the lower bound) and a period of time T_p_ for the hyperpolarization phase in neural models, such as the HH. These parameters drive the switching of the integration step size from global to local and vice versa.

**Figure 3:**
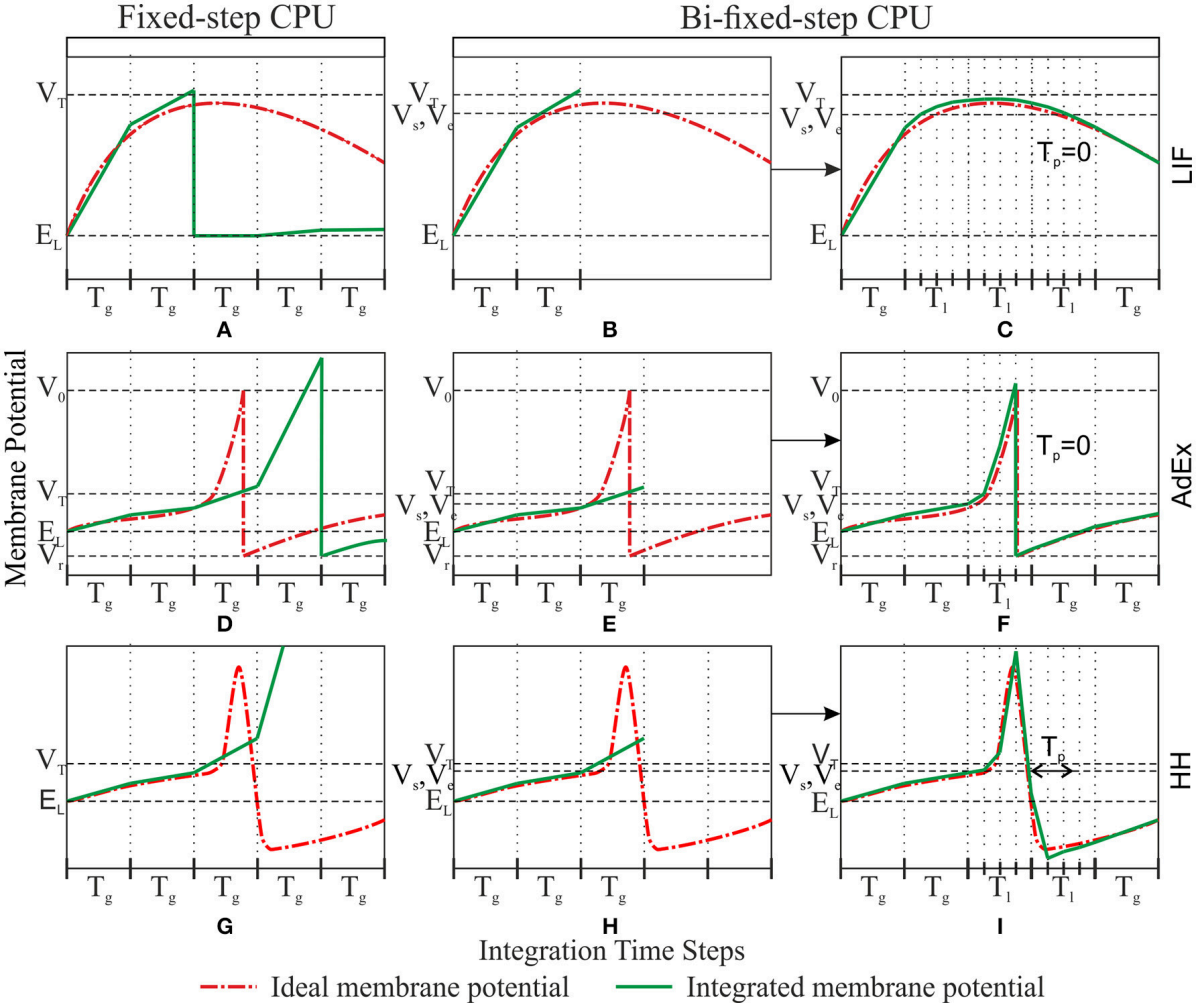
**Comparison between fixed-step and bi-fixed-step integration methods in CPU for LIF, AdEx, and HH models**. Each row shows the ideal membrane potential and the integrated membrane potential for a LIF, AdEx, and HH model, respectively. The left-hand column **(A,D**, and **G)** shows the fixed-step integration results. The central and right-hand columns show the bi-fixed-step integration results. The central column **(B,E**, and **H)** shows the moment when the membrane potential overpasses the threshold V_s_. The last integrated result is then discarded and the integration step size is scaled down to T_l_. The right-hand column **(C,F** and **I)** shows the moment when the membrane potential underpasses the threshold V_e_, a time > T_p_ has elapsed and the integration step size is then scaled up to T_g_.

This method starts the integration process of a certain group of neurons using the global integration step size T_g_ for each neuron. The membrane potential of each neuron is then compared with the threshold value V_s_ after each global integration step. When the resulting membrane potential is >V_s_, the integration result is discarded and the integration step size is scaled down to the local step size T_l_ just for that neuron. The integration process is reinitialized using the new local step size. This local step size is maintained until the membrane potential decays to V_e_ and a certain period of time T_p_ has passed. Once this double condition is filled, the integration step size is re-scaled up to the global step size T_g_ (see Figure 2A for a complete workflow diagram). Figure 3 shows an example of this adaptation mechanism over the LIF, AdEx, and HH models.

The state variables, in most neuron models, usually present a slow evolution during simulation time (very low velocity gradient). It is at the spike phase when these state variables evolve faster (very high velocity gradient). By using a V_s_ threshold lower than the actual “functional” spike threshold we are able to predict a spike phase beforehand. The bi-fixed-step integration method uses this prediction to reduce the integration step size before an eventual spike phase. T_g_ extra period sets the hyperpolarization time after the spike generation. During T_g_, the state variables present very high velocity gradients and, therefore, reduced integration step sizes are to be maintained (e.g., T_g_ can be used to properly integrate the hyperpolarization phase of HH models after the depolarization phase).

This method is easily parallelizable in CPU and can be managed with just one integration event, as in fixed-step integration methods. Additionally, it can outperform the simulation of complex neuron model with stiff equations thanks to its adaptation mechanism, as in variable-step integration methods.

##### Bi-fixed-step integration method for differential equation solvers in GPU

GPU hardware requires all the simulated neurons of a neural model to run exactly the same code at the same time. Additionally, these neurons must also access the RAM memory following a concurrent scheme. As reported in Naveros et al. (2015), the synchronization period and transference of data between CPU and GPU processors account for most of the performance losses in a hybrid CPU-GPU neural simulation. To minimize these losses, CPU and GPU processors are synchronized at each T_g_ global integration step time. Then the GPU integrates all its neurons using the local integration step T_l_. After the integration process, the GPU reports to the CPU which neurons fired a spike (see Figure 2B for a complete workflow diagram).

This method is easily parallelizable in GPU and can be managed with just one integration event, as in fixed-step integration methods. Additionally, a short local step (T_l_) can accurately compute the simulation of complex neuron model with stiff equations whereas a large global step (T_g_) reduces the number of synchronizations and data transferences between CPU and GPU processors and increases the overall performance. This bi-fixed-step integration method is suited to comply with hybrid CPU-GPU platforms since it maximizes the GPU workload and minimizes the communication between both processors.

To sum up, EDLUT can now operate with time-driven neuron models that can use different fixed-step or bi-fixed-step integration methods for both the CPU and GPU platforms. The following differential equation solvers have been implemented using fixed-step integration methods: Euler, 2nd and 4th order Runge-Kutta, and 1st to 6th order Backward Differentiation Formula (BDF). The following differential equation solvers have been implemented using bi-fixed-step integration methods: Euler, 2nd and 4th order Runge-Kutta, and 2nd order Backward Differentiation Formula (BDF). This last differential equation solver implements a fixed-leading coefficient technique (Skeel, 1986) to handle the variation of the integration step size.

In this paper, we have only evaluated the simulation accuracy and computational performance of 4th order Runge-Kutta solvers in both fixed-step and bi-fixed-step integration methods in both CPU and GPU platforms. Table 1 shows the integration parameters used by each neuron model for each integration method.

**Table 1:** **Summary of parameters for event-driven and time-driven simulation techniques for LIF, AdEx, and HH models**.

	**LIF**	**AdEx**	**HH**
Synchronization period (ms)	1.0	1.0	1.0
Fixed-step size (ms)	0.5	0.5	1.0/15.0
Global fixed-step size (ms)	1.0	1.0	1.0
Local fixed-step size (ms)	0.25	0.25	1.0/15.0
Threshold V_s_ (mV)	−53.0	−50.0	−57.0
Threshold V_e_ (mV)	−53.0	−50.0	−57.0
Period T_p_ (ms)	0.0	0.0	1.0

### Test-bed experiments

We have adapted the benchmark proposed by Brette et al. (2007) as our initial neural network setup for our experiments. The initial setup consists of 5000 neurons divided into two layers. The first layer (input layer) holds 1000 excitatory neurons and it just conveys the input activity to the second layer. The second layer consists of 4000 neurons where 80% are excitatory neurons and the remaining 20% are inhibitory neurons. The neurons at this 2nd layer are modeled as LIF, AdEx or HH models.

Each second-layer neuron is the target of 10 randomly chosen neurons from the first layer. Each second-layer neuron is also the target of eighty randomly chosen neurons from the same layer, following a recurrent topology. All these synapses include a 0.1 ms propagation delay. This neural network topology is summarized in Table 2.

**Table 2:** **Summary of cells and synapses implemented**.

**Initial Network Configuration**	**Pre-synaptic cell (number)**	**Post-synaptic cell (number)**	**Number of synapses**	**Number of excitatory synapses**	**Number of inhibitory synapses**
	Input layer (1,000) input neurons	2nd layer (4,000) LIF, AdEx, or HH neurons	40,000	40,000 (7 nS)	
	2nd layer (4,000) LIF, AdEx, or HH neurons	2nd layer (4,000) LIF, AdEx, or HH neurons	320,000	256,000 (0.5 nS)	64,000 (2.5 nS)

*Initial values providing the reference framework for comparisons in subsequent experiments*.

The input activity supplied to each input neuron is randomly generated using a Poisson process with exponential inter-spike-interval distribution and mean frequency of 5 Hz. This input activity generates a mean firing rate activity of 10 Hz in the second layer.

We measured the simulation accuracy and computational performance of the aforementioned integration methods over three different neural models (LIF, AdEx, and HH). These neural models are simulated using two different dynamic evaluation techniques: event-driven and time-driven techniques.

Within event-driven dynamic evaluation techniques, four different event-driven integration methods are applied:

- Direct event-driven integration methods.
- Combined event-driven integration methods.
- Synchronous event-driven integration methods.
- Combined synchronous event-driven integration methods.

Within time-driven dynamic evaluation techniques, two different integration methods implementing a 4th order Runge-Kutta differential equation solver are applied in both CPU and GPU platforms:

- Fixed-step integration methods.
- Bi-fixed-step integration methods.

To properly compare all these integration methods, we studied the simulation accuracy that each can offer. We used the van Rossum distance (Van Rossum, 2001) with a tau of 1 ms (maximum size of integration step periods for event-driven methods and synchronization periods for even-driven methods) as a metric of accuracy. We use this metric to compare a reference activity file and a tested activity file (both files containing the spike time stamps associated to all the spikes emitted by the neural network units). The reference activity files are obtained using a time-driven simulation technique running in CPU with a fixed-step integration method using a 4th order Runge-Kutta solver and a fixed 1 μs integration step size for each neural model (LIF, AdEx, and HH). The tested activity files are obtained using the mentioned integration methods of the two different dynamic evaluation techniques (event-driven and time-driven) for each neural model (LIF, AdEx, and HH).

Additionally, we wanted to study the computational performance of each integration method. We measured the execution time spent by each integration technique over a set of four different experiments that simulate 1 s of neural activity. These four experiments characterize the computational performance of the mentioned integration methods.

The hardware running these Benchmark analyses consists of an ASUS Z87 DELUXE mother board, an Intel Core i7-4,770k processor, 32 GB of DDR3 1,333 MHz RAM memory, and a NVIDIA GeForce GTX TITAN graphic processor unit with 6144 MB RAM memory GDDR5 and 2,688 CUDA cores. The compilers used are those that are integrated in visual studio 2008 together with CUDA 6.0.

All the experiments are CPU parallelized by using two OpenMP threads as described in a previous paper (Naveros et al., 2015). These threads parallelize the spike generation and propagation for the event-driven and time-driven models. The neural dynamic computation of the event-driven and time-driven models in CPU is also parallelized by using the two OpenMP threads. The neural dynamic computation of the time-driven models in GPU is parallelized by using all the GPU cores.

#### Simulation parameter analyses

We quantified the effects of using different simulation techniques on the simulation accuracy and the computational performance. In particular, we measured the impact of scaling the look-up table size and the synchronization time-period for event-driven techniques. For time-driven techniques, we measured the impact of scaling the integration time-step sizes.

For these analyses, our initial neural network setup is modified as defined in Table 3. A third neural layer replicating the second layer properties is inserted and the recurrent topology is modified. The 2nd and the 3rd layer are now interconnected by those synapses from the 2nd layer that were previously modeling the recurrent topology of our initial setup. This initial recurrent topology rapidly propagated and increased small errors through the recurrent synapses. Under these circumstances, accuracy cannot be properly measured. Adding this 3rd layer allowed us to circumvent this problem and better evaluate the accuracy degradation in a well-defined experiment.

**Table 3:** **Summary of cells and synapses implemented for parameter analysis experiment (accuracy and performance)**.

**Network Configuration**	**Pre-synaptic cell (number)**	**Post-synaptic cell (number)**	**Number of synapses**	**Number of excitatory synapses**	**Number of inhibitory synapses**
	Input layer (1,000) input neurons	2nd layer (4,000) LIF, AdEx, or HH neurons	40,000	40,000 (7 nS)	
	Input layer (1,000) input neurons	3rd layer (4,000) LIF, AdEx, or HH neurons	40,000	40,000 (7 nS)	
	2nd layer (4,000) LIF, AdEx or HH neurons	3rd layer (4,000) LIF, AdEx, or HH neurons	320,000	256,000 (0.5 nS)	64,000 (2.5 nS)

We stimulated this new setup with five different input patterns generated using a Poisson process with exponential inter-spike-interval distribution and mean frequency of 5 Hz. We measured the simulation accuracy of the 3rd neural layer by applying the van Rossum distances as previously explained. Thus, we were able to evaluate the effect of the synchronization period over accuracy when several layers of synchronous event-driven models are interconnected. Similarly, we also evaluated the effect of the integration step size over accuracy when several layers of time-driven models are interconnected. The computational performance is given by the mean execution time that the new setup spends in computing 1 s of simulation when it is stimulated with the five different input activity patterns.

#### Scalability analyses

We quantified the effects of scaling up the number of neurons within our initial setup over the computational performance. In particular, we measured the impact of scaling up the second layer size for the event-driven and time-driven techniques proposed.

For these analyses, our initial neural network setup is modified as defined in Table 4. Nine different variations over our initial setup are simulated. The 2nd layer is geometrically scaled up from 1,000 to 256,000 by a common ratio and scale factor of *r* = 2 and *a* = 1,000, respectively (number of neurons = *a*·*r*^*n*^, where *n* ϵ [0, 8]).

**Table 4:** **Summary of cells and synapses implemented for neural network scalability experiment**.

**Network Configuration**	**Pre-synaptic cell (number)**	**Post-synaptic cell (number)**	**Number of synapses**	**Number of excitatory synapses**	**Number of inhibitory synapses**
	Input layer (1,000) input neurons	2nd layer (from 1,000 to 256,000) LIF, AdEx, or HH neurons	From 10,000 to 256,0000	From 10,000 to 2,560,000 (7 nS)	
	2nd layer (from 1,000 to 256,000) LIF, AdEx, or HH neurons	2nd layer (from 1,000 to 256,000) LIF, AdEx, or HH neurons	From 80,000 to 20,480,000	From 64,000 to 16,384,000 (0.5 nS)	From 16,000 to 4,096,000 (2.5 nS)

#### Input activity analyses

We quantified the effects of scaling up the input activity levels over the computational performance. In particular, we measured the impact of scaling up our neural network mean-firing rate through different input activity levels for the event-driven and time-driven techniques proposed.

For these analyses, our initial neural network setup is modified as defined in Table 5. Fifteen different input activity levels scaled up from 2 to 16 Hz stimulate our neural network. These input activity levels generate mean firing rates in the second neural layer of between 2 and 40 Hz.

**Table 5:** **Summary of cells and synapses implemented for neural network input activity scaling (input average firing rate) experiment**.

**Network Configuration**	**Pre-synaptic cell (number)**	**Post-synaptic cell (number)**	**Number of synapses**	**Number of excitatory synapses**	**Number of inhibitory synapses**
	Input layer (1,000) input neurons	2nd layer (16,000) LIF, AdEx, or HH neurons	160,000	160,000 (7 nS)	
	2nd layer (16,000) LIF, AdEx, or HH neurons	2nd layer (16,000) LIF, AdEx, or HH neurons	1,280,000	1,024,000 (0.5 nS)	256,000 (2.5 nS)

#### Connectivity analyses

We quantified the effects of scaling up the number of synapses over the computational performance. In particular, we measured the impact of increasing the number of recurrent synapses for the event-driven and time-driven techniques proposed.

For these analyses, our initial neural network setup is modified as defined in Table 6. The number of recurrent synapses are geometrically scaled up from 10 to 1,280 by a common ratio and scale factor of *r* = 2 and *a* = 10, respectively (number of recurrent synapses = *a*·*r*^*n*^, where *n* ϵ *[0, 7])*. Maintaining the mean firing rate in the second neural layer at approximately 10 Hz requires the recurrent synaptic weights to be adapted depending on the number of recurrent synapses. The initial neural network maintains 80 synapses as in the previous cases (the weights of excitatory and inhibitory synapses are set to 0.5 and 2.5 nS, respectively). Neural networks with a lower number of synapses [10, 40] require the synaptic weights to remain the same. Neural networks with larger number of synapses [160, 1280] require the synaptic weights to be divided by the common ratio *r* = 2 in each iteration (the weights of excitatory and inhibitory synapses are ranged from 0.25 to 0.03125 and from 1.25 to 0.15625, respectively).

**Table 6:** **Summary of cells and synapses implemented for neural network interconnection scalability experiment**.

**Network Configuration**	**Pre-synaptic cell (number)**	**Post-synaptic cell (number)**	**Number of synapses**	**Number of excitatory synapses**	**Number of inhibitory synapses**
	Input layer (1000) input neurons	2nd layer (16,000) LIF, AdEx, or HH neurons	160,000	160,000 (7 nS)	
	2nd layer (16,000) LIF, AdEx, or HH neurons	2nd layer (16,000) LIF, AdEx, or HH neurons	From 160,000 to 20,480,000	From 128,000 to 16,384,000 (from 0.5 to 0.03125 nS)	From 32,000 to 4,096,000 (from 2.5 to 0.15625 nS)

## Results

This section shows the results obtained by the four test-bed experiments described in the methods section. Each experiment evaluated eight different neural dynamic simulation techniques over LIF, AdEx, and HH neuron models in terms of accuracy and/or performance (see Methods). Thus, we evaluated how the proposed simulation techniques perform with neural models of different mathematical complexity.

### Simulation parameter results: the look-up table, synchronization period and integration step size implications

In this experiment, we computed the neural network defined in Table 3 using five different input spike patterns with a Poisson process and mean firing rate of 5 Hz. We measured the simulation accuracy over the third neural layer and the computational performance over the whole simulation time when the look-up table size, the synchronization period, or the integration step size are scaled up.

#### Look-up table size implications

As previously stated, the look-up table size directly affects the neural model simulation accuracy and computational performance for event-driven simulation techniques. This is of special importance for neural models with high mathematical complexity. The number of state variables in a model determines the number of look-up tables and their dimensions. Since the number of state variables is given by the neural model (LIF, AdEx, or HH), the granularity level of each look-up table dimension is the only independent parameter that can be freely selected to adjust the look-up table size. The more complex the model is, the lower granularity levels that are required to keep the look-up table size manageable (HH granularity level <AdEx granularity level <LIF granularity level). Consequently, the higher the complexity of the neural models is, the lower accuracy that is obtained when the total look-up table sizes are fixed.

Here, we have evaluated four pre-compiled look-up tables with different levels of granularity for each neural model. In subsequent experiments, the event-driven models will use the largest look-up tables to keep the highest possible level of accuracy.

**Figure 4:**
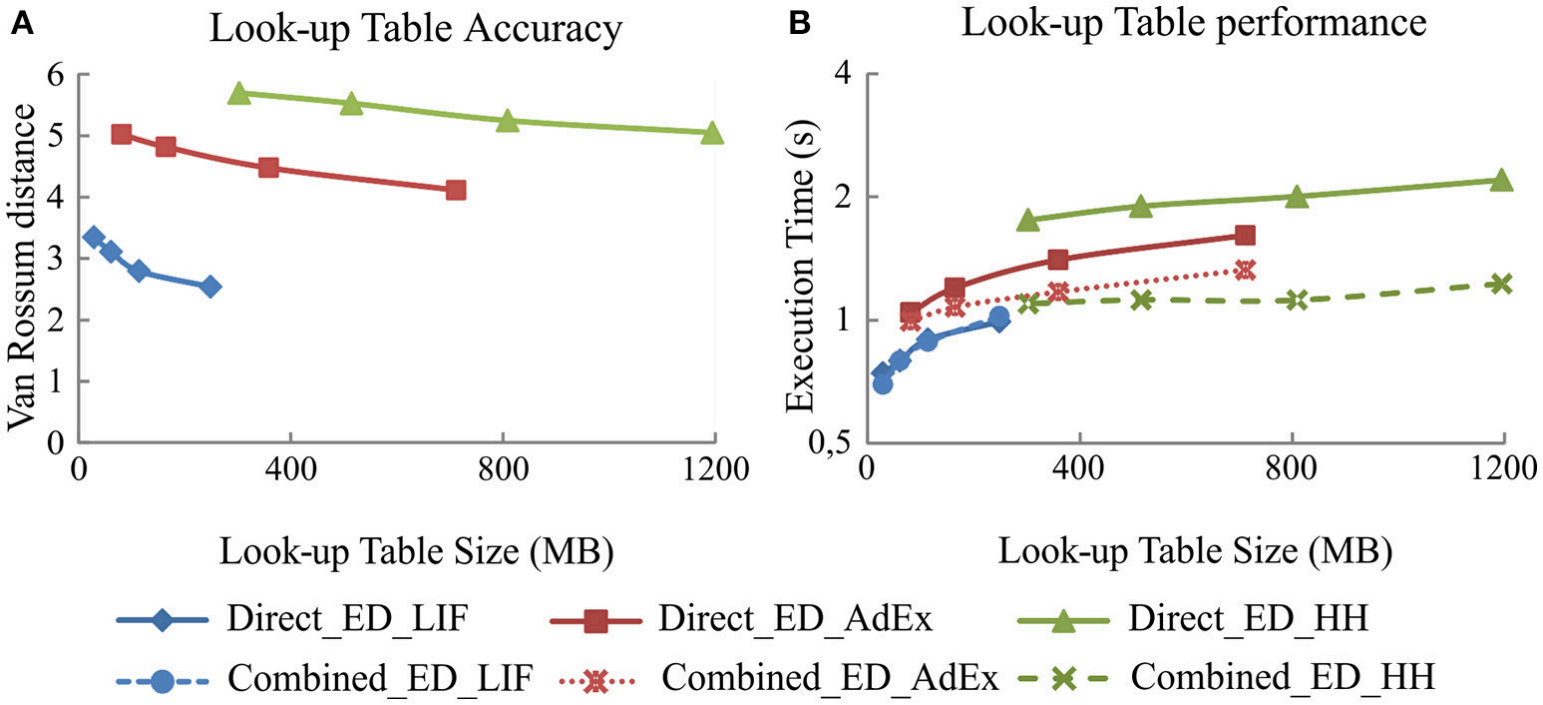
**Simulation accuracy and computational performance for direct and combined event-driven integration methods. (A)** Mean simulation accuracy obtained with direct and combined event-driven integration methods depending on the look-up table sizes for five different input spike patterns. **(B)** Computation time spent by direct and combined event-driven integration methods in running 1 s of simulation over five different input spike patterns (averaged). Four different look-up table sizes for each neural model are used. The neural network defined in Table 3 is simulated using both integration methods over LIF, AdEx, and HH models. The standard deviation of simulation accuracy and computational performance obtained is negligible; we only represent the mean values. Both integration methods present identical accuracy results.

Figure 4 shows the simulation accuracy and computational performance of direct and combined event-driven integration methods with respect to the four sets of look-up tables with different levels of granularity for each of our three neural models (LIF, AdEx, and HH). As shown, the larger the look-up table size is, the higher the accuracy (smaller van Rossum distances with respect to the reference simulation pattern; Figure 4A) but at the cost of a worse performance (higher execution times; Figure 4B). The simulation accuracy for both integration methods (direct and combined) remains the same since the look-up table recombination of the second integration method does not affect the neural dynamics. The simulation accuracy for one of these integration methods is, therefore, representative for both in the plots of Figure 4.

Figure 4B also compares the computational performance of direct and combined event-driven integration methods. The more mathematically complex the neural model is, the more look-up tables can be combined and the higher the computational performance results of combined event-driven integration methods with respect to the direct ones.

#### Synchronization period size implications

**Figure 5:**
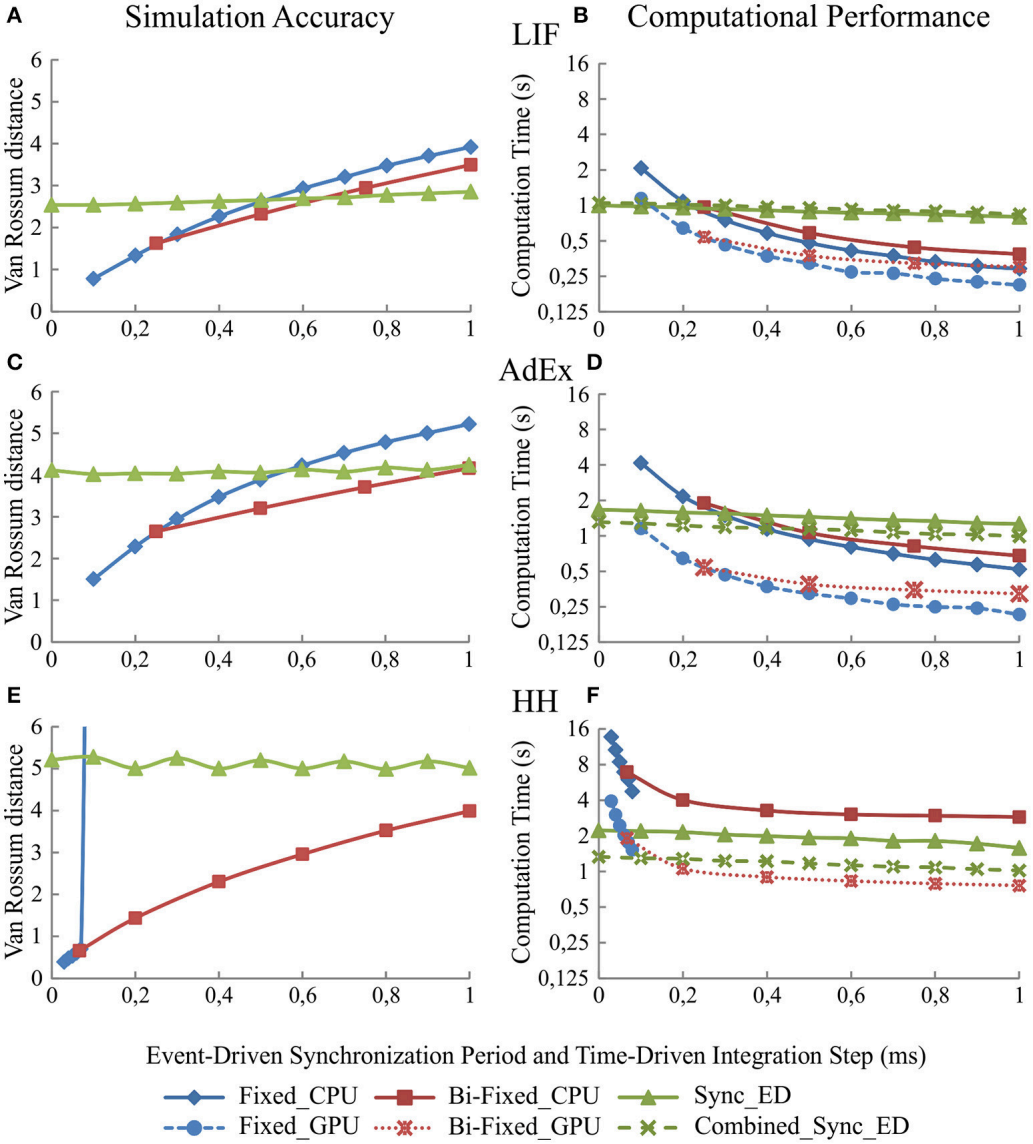
**Simulation accuracy and computational performance of synchronous and combined synchronous event-driven integration methods depending on the synchronization periods**. Simulation accuracy and computational performance of fixed-step and bi-fixed-step integration methods in CPU and GPU platforms depending on the integration step size. One-second simulation of the neural networks defined in Table 3 is shown. Five different input spike patterns of 5 Hz that generate mean firing rate activities of 10 Hz in the third neural layer are used. The left-hand column **(A,C**, and **E)** of the panel shows the simulation accuracy and the right-hand column **(B,D**, and **F)** the computational performance obtained by the synchronous and combined synchronous event-driven integration methods depending on the synchronization period for LIF, AdEx, and HH models, respectively (the synchronization period is plotted over x-axis). Both columns also show the simulation accuracy and computational performance of fixed-step and bi-fixed-step integration methods in CPU and GPU platforms depending on the integration steps (the global integration step size of fixed-step and bi-fixed-step integration methods are plotted over x-axis. The local step sizes for bi-fixed-step integration methods are 0.25 ms for LIF and AdEx models and 1/15 ms for HH model). The standard deviation of the simulation accuracy and the computational performance obtained is negligible; we only represent the mean values. Synchronous and combined synchronous event-driven integration methods present identical accuracy results. CPU and GPU time-driven integration methods present almost identical accuracy results. The stiffness of HH model constrains the maximum step size that fixed-step integration methods can use. Beyond this step size, the differential equations cannot properly be integrated for this model.

The synchronization period of synchronous and combined synchronous event-driven integration methods affects the neural model simulation accuracy and the computational performance. As in the previous case, the simulation accuracy of both integration methods remains the same since the look-up table recombination does not affect the neural dynamics. The simulation accuracy for one of these integration methods is, therefore, representative for both in plots of Figure 5.

Both integration methods minimize the number of spike predictions when the input activity is synchronous (see methods). Adjusting the step size of the synchronization period in the second neural layer allows us to control the synchronicity of the input activity driven toward the third neural layer (see Table 3).

Figure 5 shows to what extent the synchronization period affects the simulation accuracy (Figures 5A,C, and E) and the computational performance (Figures 5B,D, and F) for each of our three neural models (LIF, AdEx, and HH), respectively. The larger the synchronization period is, the higher the computational performance (shorter execution times). Regarding simulation accuracy, event-driven methods are comparable in accuracy to time-driven methods for LIF and AdEx models. Conversely, event-driven methods present larger accuracy errors for the HH model due to RAM capacity limitations (huge look-up tables would be required to achieve similar accuracy rates).

#### Integration step size implications

Likewise, simulation accuracy and computational performance in time driven simulation techniques using fixed-step and bi-fixed-step integration methods are tightly related to the integration step sizes. The simulation accuracy for both methods in CPU and GPU platforms is almost identical. For the sake of readability, we only show the accuracy results of CPU methods in Figure 5. Figure 5 shows to what extent the decrease of the integration step size affects the simulation accuracy and the computational performance. The smaller the integration step sizes are, the more accurate the results that are obtained (Figures 5A,C, and E) but at the cost of slower simulations (Figures 5B,D, and F). When comparing the computational performance of fixed-step and bi-fixed-step integration methods in both platforms, CPU and GPU, it is demonstrated that the more complex the neural model is, the better performance results that are obtained by the bi-fixed-step methods with respect to the fixed-step ones.

### Scalability results: implications when increasing the number of network units

This section studies the computational performance for the event-driven and time-driven simulation techniques when the neural network size is scaled up. We have measured the computational performance of our two different dynamic evaluation techniques when they are applied to different neural network sizes (Table 4) under equal input activity patterns (a set of random input patterns with 5 Hz mean frequency).

**Figure 6:**
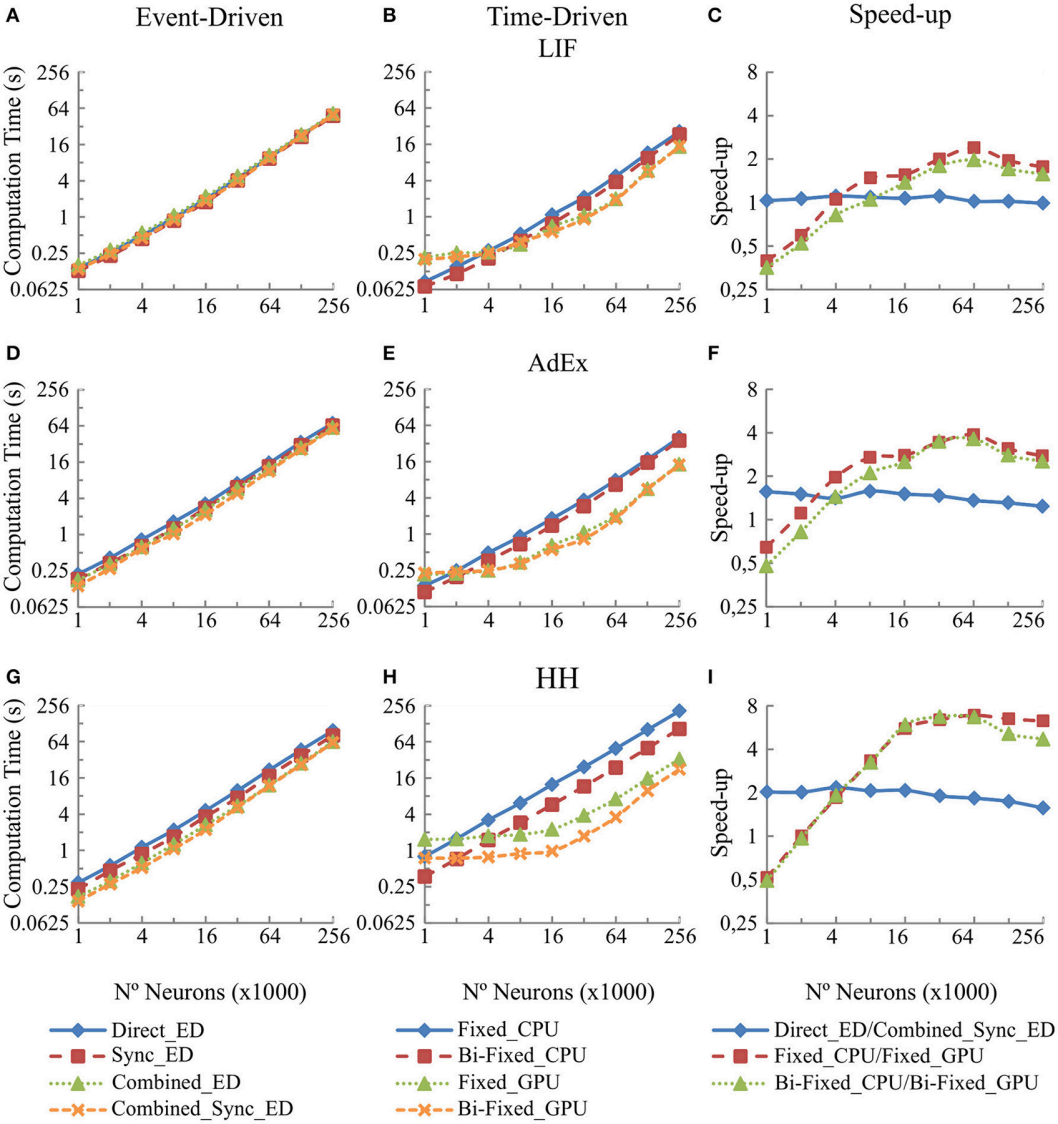
**Computational performance obtained from the eight integration methods proposed for LIF, AdEx and HH neural models depending on the number of neurons within the second neural layer**. One-second simulation of the neural networks defined in Table 4 is shown. A mean input activity of 5 Hz that generates a mean firing rate activity of 10 Hz in the second neural layer is used. The left-hand column **(A,D**, and **G)** of the panel shows the computational performance of the four event-driven integration methods (direct, combined, synchronous, and combined synchronous event-driven methods) for LIF, AdEx, and HH neural models, respectively. The central column **(B,E**, and **H)** of the panel shows the computational performance of the four time-driven integration methods (fixed-step and bi-fixed-step integration methods in both CPU and GPU platforms) for the same three neural models. The right-hand column **(C,F**, and **I)** of the panel shows the speed-up achieved by the combined synchronous event-driven methods, the fixed-step and bi-fixed-step integration methods in GPU respect to the direct event-driven methods, the fixed-step and bi-fixed-step integration methods in CPU for the same three neural models.

Figure 6 shows in the column on the left (Figures 6A,D, and G) the computational performance of our four event-driven integration methods (direct, combined, synchronous, and combined synchronous event-driven integration methods) for LIF, AdEx and HH neural models, respectively. The central column (Figures 6B,E, and H) shows the computational performance of our four time-driven simulation methods (fixed-step and bi-fixed-step integration methods in both CPU and GPU platforms) for the same three neural models. The column on the right (Figures 6C,F, and I) shows the speed-up achieved by the combined synchronous event-driven methods, the fixed-step and bi-fixed-step integration methods in GPU with respect to the direct event-driven methods, the fixed-step and bi-fixed-step integration methods in CPU for the same three neural models.

The six CPU methods (direct, combined, synchronous, and combined synchronous event-driven integration methods as well as fixed-step and bi-fixed-step integration methods) present a linear behavior. The computation time linearly increases with the number of neurons. Similarly, the two GPU integration methods (fixed-step and bi-fixed-step integration methods) perform linearly with the number of neurons (the computation time increases with the number of neurons). However, when the number of neurons to be simulated is under a certain boundary, the time spent in the synchronization and transference of data between CPU and GPU processors dominates over the neural computation time. In this case, the speed-up of GPU methods with respect to the CPU ones decreases, as shown in Figure 6, right column.

### Input activity results: implication when increasing the mean firing activity

This section studies the computational performance of the event-driven and time-driven simulation techniques as the mean firing activity of the neural network increases. The neural network described in Table 5 has been simulated using fifteen different input activity patterns whose mean firing rate frequency ranges from 2 to 16 Hz.

**Figure 7:**
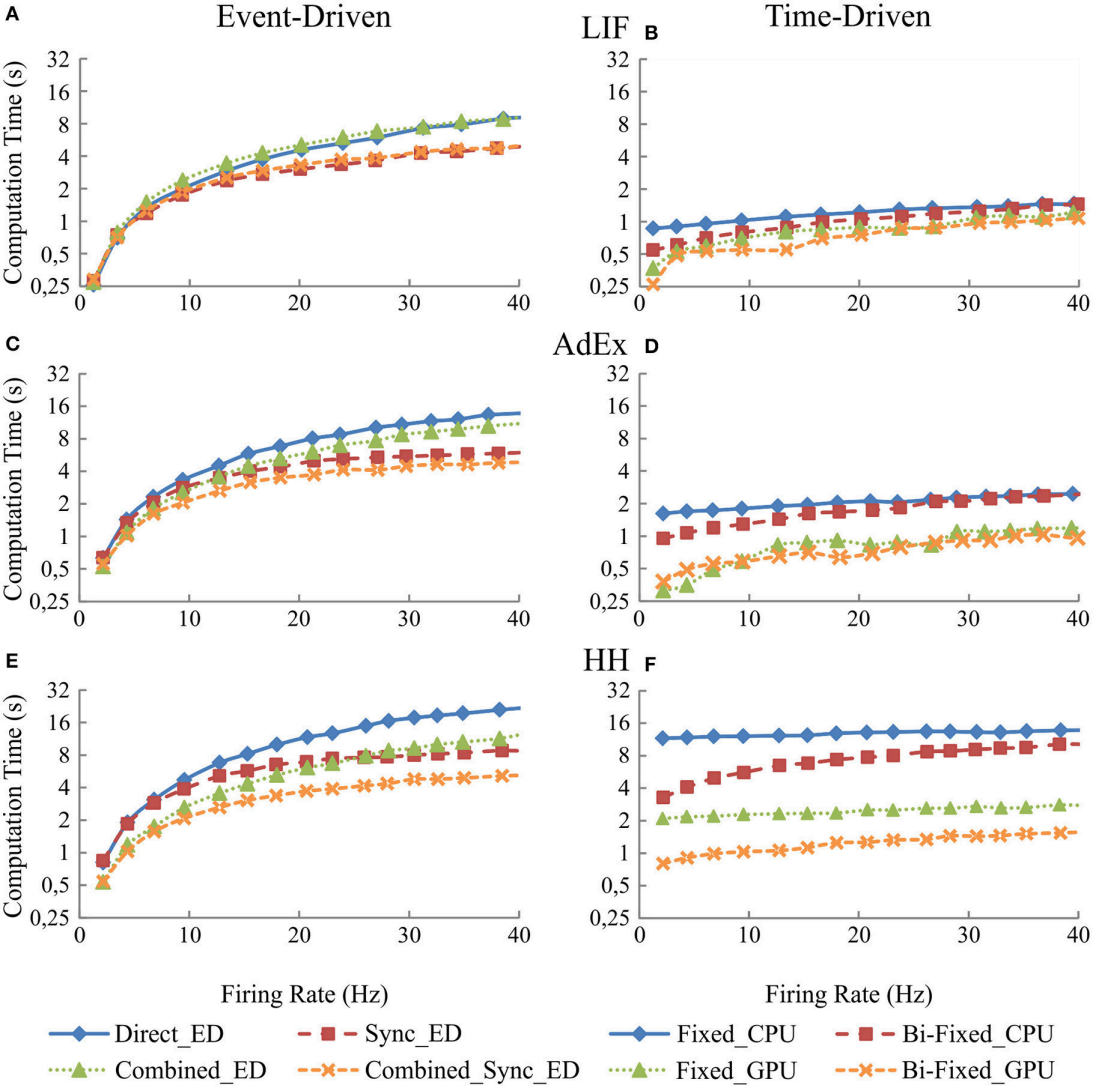
**Computational performance obtained from the eight simulation methods proposed for LIF, AdEx, and HH neural models depending on the mean firing rate activity in the second neural layer**. One-second simulation of the neural networks defined in Table 5 is shown. The neural input activity ranges from 1 to 10 Hz on average. The mean neural activity obtained at the second layer is plotted over x-axis. The left-hand column **(A,C**, and **E)** of the panel shows the computational performance of the four event-driven integration methods (direct, synchronous, combined, and combined synchronous event-driven integration methods) for LIF, AdEx, and HH neural models, respectively. The right-hand column **(B,D**, and **F)** of the panel shows the computational performance of the four time-driven integration methods (fixed-step and bi-fixed-step integration methods in both CPU and GPU platforms) for the same three neural models.

Figure 7 shows in the column on the left (Figures 7A,C, and E) the computational performance of our four event-driven integration methods (direct, combined, synchronous, and combined synchronous event-driven integration methods) for LIF, AdEx, and HH neural models, respectively. The column on the right (Figures 7B, D, and F) shows the computational performance of the four time-driven simulation methods (fixed-step and bi-fixed-step integration methods in both CPU and GPU platforms) for the LIF, AdEx, and HH neural models, respectively.

Figure 7 clearly shows how event-driven schemes are sensitive to the level of input activity, whilst the impact of the input activity on time-driven integration methods is marginal. When comparing amongst event-driven integration methods, the results clearly show the improvements of the combined and synchronous integration methods. The slope of the result series (i.e., the impact of the average activity on the simulation performance) decreases when these integration methods are adopted in the simulation scheme. When comparing amongst time-driven methods, the improvements of using bi-fixed-step methods (leading to 2-fold performance levels compared to fixed-step methods) and GPU as co-processing engine (leading to 5-fold performance levels compared to CPU approaches) are also clear in the obtained results. Amongst the four time-driven integration methods proposed, the bi-fixed-step method in CPU is the most severely affected by the increasing of the mean firing activity within the neural network. The overhead time spent in deploying the adaptation mechanism (see methods) makes these integration methods unadvisable for scenarios where the neural network presents very high levels of constant firing activity.

### Connectivity results: implications when increasing the number of synapses in the recurrent topology

This section studies the computational performance for the event-driven and time-driven simulation techniques as the number of synapses in the recurrent topology of our neural network increases. The neural network described in Table 6 has been simulated using a random input activity with a mean firing rate of 5 Hz.

**Figure 8:**
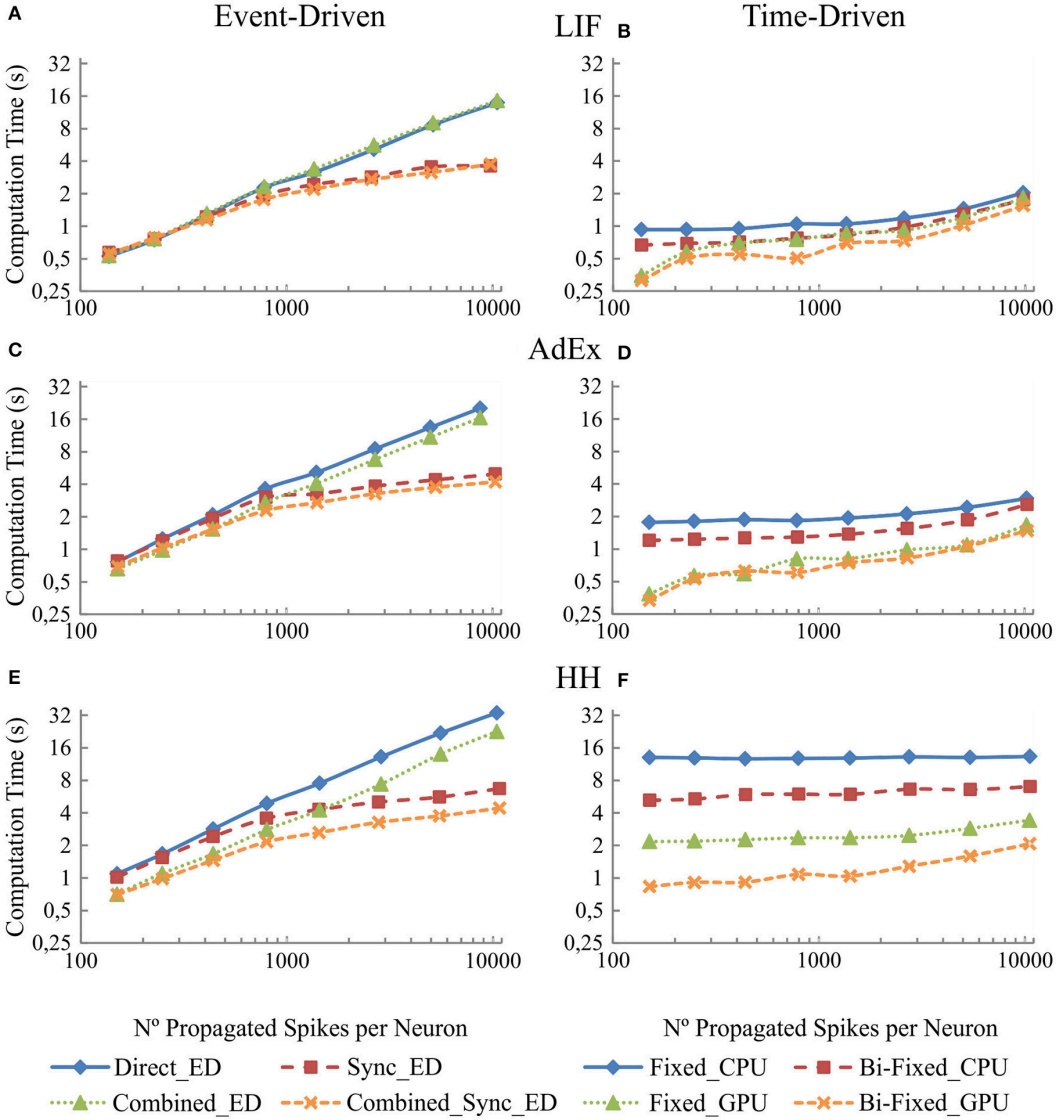
**Computational performance of the eight simulation methods proposed for LIF, AdEx, and HH neural models depending on the number of propagated spikes that are determined by the number recurrent synapses**. One-second simulation of the neural networks defined in Table 6 is shown. The number of recurrent synapses is geometrically scaled up (10, 20, 40, 80, 160, 320, 640, and 1,280). A mean input activity of 5 Hz is used. This input activity generates a mean firing rate activity of between 8 and 12 Hz within the second neural layer. The number of propagated spikes increments proportionally with the number of synapses (number of propagated spikes = number of internal spikes · number of recurrent synapses). The mean number of propagated spikes that arrives to the second layer is plotted over x-axis. The left-hand column **(A,C**, and **E)** of the panel shows the computational performance of the four event-driven integration methods (direct, synchronous, combined, and combined synchronous event-driven integration methods) for LIF, AdEx, and HH neural models, respectively. The right-hand column **(B,D**, and **F)** shows the computational performance of the four time-driven integration methods (fixed-step and bi-fixed-step integration methods in both CPU and GPU platforms) for the same three neural models.

Figure 8 shows in the column on the left (Figures 8A,C, and E) the computational performance of our four event-driven integration methods (direct, combined, synchronous, and combined synchronous event-driven integration methods) for LIF, AdEx, and HH neural models, respectively. The column on the right (B, D, and F) shows the computational performance of our four time-driven integration methods (fixed-step and bi-fixed-step integration methods in both CPU and GPU platforms) for the LIF, AdEx, and HH neural models, respectively. The firing rate activity remains quite stable (between 8 and 12 Hz), although the number of propagated spikes increases due to the higher number of synapses. The computation time (the measured variable) depends on the computational workload. This workload, in turn, depends on the number of internal spikes and recurrent synapses (number of propagated spikes = number of internal spikes · number of recurrent synapses). The number of propagated spikes is plotted in x-axis instead of the number of recurrent synapses to better compare the computation time of all the simulation methods under equivalent neural activity conditions. Each mark in Figure 8 corresponds to a number of recurrent synapses (10, 20, 40, 80, 160, 320, 640, and 1280) since this is the parameter that can be directly set in the network definition and thus in the simulation experiment.

The simulation performance in event-driven integration methods significantly decreases as the number of propagated spikes increases. Nevertheless, we can see a significant improvement when synchronous event-driven integration methods are used since they are optimized for computing higher levels of synchronous activity. Conversely, the simulation performance in time-driven integration methods suffers little direct impact as the number of propagated spikes increases. The results show the improvement achieved with the bi-fixed-step integration methods either with or without GPU co-processing.

When comparing amongst event- and time-driven methods, GPU time-driven methods have the best-in-class performance (see Figures 7, 8). Incremental levels of input activity cause an incremental number of propagated spikes thus favoring GPU time-driven methods. On the other hand, tend-to-zero input activity levels favor event-driven methods. The event-driven performance obtained under these low input activities is usually equal to or better than GPU time-driven performance.

## Discussion

Throughout this paper, different neural dynamic evaluation techniques are developed. Within the event-driven methods: the combined integration methods based on the combination of look-up tables and the synchronous integration methods based on the optimization of processing synchronous activities. These two integration method are clear improvements with respect to previously described event-driven neural dynamic evaluation techniques (Ros et al., 2006a). As far as the time-driven methods are concerned, the bi-fixed-step integration methods and the CPU-GPU co-processing significantly increase the performance of time-driven neural dynamic evaluation techniques.

The quality level of each proposed integration method is given in terms of neural accuracy and computational performance when simulating three neural models of incremental mathematical complexity (LIF, AdEx, and HH). These neural models are set up (Table 7) for reproducing similar activity patterns. All the simulation methods shall provide similar accuracy results to make them comparable. Fixed-step and bi-fixed-step time-driven integration methods for LIF and AdEx models are set up (Table 1) for obtaining similar accuracy results than event-driven methods (Figure 5). LIF and AdEx models are compiled in look-up tables of 249 and 712 MB, respectively (Figure 4).

**Table 7:** **Summary of parameters for LIF, AdEx and HH neural models**.

**LIF**		**AdEx**		**HH**	
C	0.19e–9 F	C	110 pF	C	120 pF
E_L_	−0.065 V	E_L_	−65 mV	E_L_	−65 mV
g_L_	10e–9 S	g_L_	10 nS	g_L_	10 nS
V_T_	−0.050 V	V_T_	−50 mV	V_T_	−52 mV
T_ref_	0.0025 s	Δ_T_	2 mV	g_Na_	20 nS
E_AMPA_	0.0 V	τ_w_	50 ms	E_Na_	50 mV
E_GABA_	−0.080 V	A	1 nS	g_Kd_	6000 nS
τ_AMPA_	0.005 s	B	9 pA	E_K_	−90 mV
τ_GABA_	0.010 s	V_r_	−80 mV	E_AMPA_	0.0 mV
		E_AMPA_	0.0 mV	E_GABA_	−80 mV
		E_GABA_	−80 mV	τ_AMPA_	5 ms
		τ_AMPA_	5 ms	τ_GABA_	10 ms
		τ_GABA_	10 ms		

The higher complexity of the HH model imposes a large storage memory capacity. An event-driven HH model with comparable accuracy levels to bi-fixed-step time-driven HH model would require up to 14 GB of storage memory capacity (estimation extrapolated from Figure 4A). In this benchmark, the HH model has been compiled in look-up tables of 1195 MB that obtain larger accuracy errors results than the equivalent time-driven methods.

### Event-driven main functional aspects

The main functional aspects in relation to the event-driven integration methods can be summarized as follows:

- The number of state variables defining a neural model represents, broadly speaking, the complexity of a neural model. When this number increases linearly, the memory requirements to allocate the pre-compiled look-up tables of the event-driven neural models increases geometrically. Thus, reducing the level of granularity of each dimension is the only way to reduce the total look-up table size, but this reduction directly affects the simulation accuracy (as shown in Figure 4A). The more complex the neural models are or the smaller the look-up table sizes are, the higher van Rossum distance values (less accuracy) that are obtained. Boundaries in accuracy and memory capacity constrain the maximum neural complexity that these event-driven techniques can handle.
- The recombination of look-up tables improves the computational performance, maintaining the simulation accuracy. Actually, the combined event-driven integration methods slightly increase the computation time when the neural model complexity increases because the neural state update process of several variables using combined look-up tables is slightly more complex than the update of just one variable. Larger look-up table sizes cause higher rates of cache failures and, therefore, losses in computational performance (see Figure 4). This means that the computational performance is more impacted by the total look-up table size than by the mathematical complexity (the number of state variables) of the neural model, although both the mathematical complexity and the look-up table size are related.
- The computation mechanism used by synchronous methods to deal with synchronous activity significantly improves the computational performance. When a synchronous event-driven neuron receives input synapses coming from other synchronous event-driven neurons or time-driven neurons, the computational performance enhancement depends on either the synchronization period or the integration step size of the previous layers. The larger the synchronization period or the integration step size of the previous layers are, the more synchronous the activity that arrives to the synchronous model and the higher performance levels with respect to the direct non-synchronized integration methods (see Figures 5–8). Regarding the simulation accuracy, the look-up tables are precompiled maintaining a certain degree of precision. A lager synchronization period only generates a negligible error in the spike generation time which, in turn, causes small oscillations in the van Rossum distance measurements (Figure 5).
- Both neural dynamic evaluation techniques (the combination of look-up tables and synchronization of activity) are simultaneously applied by the combined synchronous event-driven method. This simulation technique outperforms the rest of event-driven techniques. This is more significant when the mathematical complexity of the neural models increases (see Figures 5–8).
- The main factor that finally constrains the computational performance of all these event-driven methods is the number of events that need to be processed. These events are mainly internal and propagated spikes (Ros et al., 2006a) that linearly increase with the neural activity. Time-driven integration methods are preferred rather than event-driven integration methods for those neural networks with high levels of neural activity (see Figures 7, 8). Conversely, there are particular cases in which the event-driven integration methods can be the best option. There are, actually, biologically realistic SNNs in which parts of their inner layers present a very low and sparse neural activity, such as the granular cells in the cerebellum (D'Angelo et al., 2016) or the mushroom bodies within the olfactory system in Drosophila (Serrano et al., 2013). The importance of these particular networks cannot be overlooked (i.e., just the granular cerebellar layer accounts for half of the neurons of the whole brain, its neurons receive between three and six input synapses with a low and very sparse activity, with most of them remaining silent and barely generating spikes). In these cases, event-driven integration methods perform better than time-driven integration methods.

### Time-driven main functional aspects

The main functional aspects in relation to the time-driven integration methods can be summarized as follows:

- Hybrid CPU-GPU integration methods perform better than CPU methods. This is specifically relevant when the mathematical complexity of the neural models increases. GPU hardware architecture performs better computing parallel tasks than CPU architecture. The computation of the neural dynamics is a pure parallelizable task and consequently, GPU-friendly. In a hybrid CPU-GPU platform, the GPU only processes the neural dynamics, whilst the spike generation and propagation are processed in the CPU. When the mathematical complexity of the neural models increases, the workload assigned to the GPU increases, whilst the workload of the CPU remains equal. For this reason, CPU-GPU neural models perform better than purely CPU neural models, especially when the mathematical complexity of the neural models increases. This increase in performance is shown in Figures 5–8.
- Bi-fixed-step integration methods outperform fixed-step integration methods for both CPU and GPU platforms when the mathematical complexity of the neural model increases (see Figures 5–8). Complex neural models usually demand small integration step sizes to better cope with the stiffness of their neural model equations during the spike shape generation. Figures 5E,F show how the maximum step size on a fixed-step integration method is constrained due to the differential equation stiffness (HH model). The adaptation mechanism used by the CPU bi-fixed-step integration methods improves the simulation performance by enlarging the simulation step size during those neural dynamic intervals out of the spike phase.
- The adaptation mechanism of the integration step size for GPU bi-fixed-step integration methods increases performance thanks to the minimization of the time spent in the synchronization and transfer of data between the CPU and GPU processors.
- Whilst CPU integration methods are better suited for small-medium groups of neurons (from one neuron to several thousands of neurons, depending on the mathematical complexity), the GPU integration methods are better suited for larger numbers of neurons (from thousands to millions of neurons). The computation time invested in the synchronization period and data transferences between CPU and GPU platforms dominates over the computation time invested in solving the neural dynamics when the number of neurons within the network is small (see Figure 6). In this case, the computational performance of the GPU integration methods reaches a plateau.
- The adaptation mechanism that the bi-fixed-step integration method uses in CPU may decrease the computational performance when the mean firing rate over the neural network is quite high. When the neural activity increases, the ratio of use between the local and global step also increases. The computational workload for the neural dynamic increases and the performance drops (see how the computation time increases in Figure 7).

### EDLUT hybrid architecture into perspective

EDLUT is a simulator mainly oriented to efficiently simulate medium-scale neural networks (tens of thousands of neurons) pursuing real time simulations. EDLUT uses point neural models, such as LIF, AdEx or HH. EDLUT information transmission relies on spike timing rather than on the particular spike shape. What matters is when the spike is emitted rather than how the spike is generated. Neurons are just means to an end needed toward understanding the behavior of the neural network behind. The neural communication mechanisms are deployed at network level at very high simulation speeds on a single multicore computer, thus facilitating real time embodiment experiments (Carrillo et al., 2008; Luque et al., 2011a,b, 2014a,b, 2016; Garrido et al., 2013a; Casellato et al., 2014; Antonietti et al., 2016). In these neurorobotic experimental set-ups the neural network and the body are coupled as a single entity.

Conversely, NEURON (Hines and Carnevale, 1997) is mainly designed for the simulation of very complex and detailed neural models. What matters here is how the spike was generated rather than when it was emitted. Understanding neurons themselves is the goal. To be as biologically plausible as possible, NEURON is conceived to deal with high levels of mathematical complexity that usually require time-driven simulation methods (either fixed- or variable-step integration methods). The computational cost here highly depends on the mathematical complexity which makes the simulation of hundreds or tens of hundreds neurons conforming a network almost computationally intractable. Using NEURON for the benchmark analysis proposed here would be out of context.

NEURON, lately, seems to be increasing its field of application toward medium- large-scale neural networks (see Lytton et al., 2016) that are comprised of highly simplified neural models (i.e., Izhikevich or the four dimensional HH models). Note that the time-driven simulation techniques here proposed may have a direct impact on NEURON if this tendency is finally consolidated.

In contrast, BRIAN (Goodman and Romain, 2009) and NEST (Gewaltig and Diesmann, 2007) are simulators often considered to be playing in the same league as EDLUT. As is the case with EDLUT, Brian claims to be mainly oriented to efficiently simulate medium-scale neural networks (tens of thousands of neurons) while NEST is designed for very large-scale neural networks (up to 1.86 billion neurons connected by 11.1 trillion synapses on the Japanese K supercomputer; Kunkel et al., 2014). These simulators mainly implement point neuron models, although some models with few compartments can be simulated. Similarly, they consider neurons to be just means to an end. They use neurons to understand the behavior of the neural network behind. Both are natively implementing time-driven simulation methods in CPU and particularly BRIAN also implements a hybrid CPU-GPU co-processing scheme for time-driven models. Having said that, the conclusions and approaches proposed in the paper regarding time-driven methods would have a direct impact on Brian and a substantial impact on NEST since CPU-GPU co-processing is still missing. The other fundamental pillar of the methodology proposed here, the event-driven scheme, is not included in BRIAN but it does exist in NEST. Whilst the event-driven EDLUT framework (originally an event-driven scheme) was adapted to also perform time-driven neural simulations (Garrido et al., 2011), the time-driven NEST framework (originally a time-driven scheme) was adapted to also perform event-driven neural simulations (Morrison et al., 2007; Hanuschkin et al., 2010). Thus, both simulators can perform combined event- and time-driven simulations. In fact, NEST proposes an event-driven method that presents similarities to our synchronous event-driven method. Both event-driven methods minimize the number of spike predictions by processing all the synchronous input spikes conjointly and thus make only one prediction.

## Conclusions

The way forward in computational neuroscience lies in the simulation of biologically plausible computational models of different nervous centers (cerebellum, inferior olive, cuneate nucleus, etc.) to better understand how the information is processed within these nervous centers. Computational neuroscience allows the study of these nervous center models without experimental restrictions using neural models that have been developed and validated according to experimental cellular data.

These nervous center models can be simulated in different conditions and circumstances to give a consistent idea about how they may operate. In many cases, these models are becoming a fundamental tool in the neuroscience hypothesis-experimentation cycle. The computational models allow researchers to test their hypotheses in simulation. This fact leads to making a better hypothesis and better experiments designed with a greater probability of success.

The road to model and simulate nervous centers has been progressively paved with increasing levels of mathematical complexity to include more and more biological features. However, this mathematical complexity comes at a computational cost (i.e., neural accuracy and computational performance). In this paper, we have proposed several new neural dynamic evaluation techniques to cope with the incremental mathematical complexity of well-known neural models (LIF, AdEx, and HH):

- The combined synchronous event-driven integration method combines the look-up tables to minimize the number of look-up table data queries needed to update the neural state variables during the simulation process. Additionally, this method also minimizes the look-up table data queries, making just one prediction about the emission of an output spike for each group of synchronous input spikes that arrive to each neuron.
- The bi-fixed-step integration method (optimized also in GPU) in which the neural dynamic equations that define the complex neural models (as HH) are accurately solved by switching between two time steps of different lengths during the simulation process.

All these integration methods, with their own pros and cons, are meant to be used concurrently to increase the computational performance when simulating heterogeneous SNNs (such as those previously studied in Naveros et al., 2015). These heterogeneous SNNs consist of several layers with different neural properties, thus trying to mimic the neural heterogeneity found in different brain regions, such as the cerebellum (D'Angelo et al., 2016; Luque et al., 2016) or the cuneate nucleus (Bologna et al., 2011). The simulation platform used in this study integrates all these neural dynamic evaluation techniques in such a way that parts of the neural network (with low and sparse activity) can be simulated efficiently with event-driven methods (which have been optimized to more efficiently deal with relatively-complex neural models and synchronous activity) and parts of the neural networks (with higher activity in terms of number of spikes) can be simulated with time-driven methods (which have been optimized with bi-fixed-step integration methods and the capability of using highly parallel hardware, such as GPU engines). See Appendix B for a simulation accuracy study of neural networks with combined event- and time-driven methods.

Choosing the most appropriate method or combination of methods for each neural center model to be simulated is a trade-off amongst three elements:

1. The neural network architecture (number of neurons, neural model complexity, number of input and output synapses, mean firing rates, etc.).
2. The hardware restrictions (number of CPU and GPU cores, RAM size).
3. The simulation requirements and target (minimizing the execution time, maximizing accuracy, etc.).

Finally, this study has been done using neural networks with medium-low connectivity ratios (from 10 to 1280 input synapses per neuron) oriented to fast simulations. However, the simulation performance results may change significantly when simulating neural networks with larger connectivity ratios (for example 10,000 input synapses per neuron). In this case the spike propagation task is usually more time consuming than the neural dynamics update task for time-driven methods. Nevertheless, as can be seen in Figure 8, our synchronous event-driven method improves its performance in relation to the direct event-driven method when the number of synapses increases.

## Author contributions

All authors listed have made substantial, direct and intellectual contribution to the work, and approved it for publication. FN, RC, JG, and NL conceived and designed the experiments. FN performed the experiments. FN, RC, NL, JG, and ER analyzed the data. FN, NL, and RC contributed reagents/materials/analysis tools. FN, NL, and ER wrote the paper.

### Conflict of interest statement

The authors declare that the research was conducted in the absence of any commercial or financial relationships that could be construed as a potential conflict of interest.
