# Corrigendum: Event- and Time-Driven Techniques Using Parallel CPU-GPU Co-processing for Spiking Neural Networks

**DOI:** 10.3389/fninf.2018.00024

**Published:** 2018-05-04

**Authors:** Francisco Naveros, Jesus A. Garrido, Richard R. Carrillo, Eduardo Ros, Niceto R. Luque

**Affiliations:** ^1^Department of Computer Architecture and Technology, Research Centre for Information and Communication Technologies, University of Granada, Granada, Spain; ^2^Vision Institute, Aging in Vision and Action Lab, Paris, France; ^3^CNRS, INSERM, Pierre and Marie Curie University, Paris, France

**Keywords:** event- and time-driven techniques, CPU, GPU, look-up table, spiking neural models, bi-fixed-step integration methods

In the original article, we neglected to acknowledge the funding from the European Commission (653019 - CEREBSENSING) to JG. The authors apologize for this oversight. This error does not change the scientific conclusions of the article in any way.

The original article was updated.

## Conflict of interest statement

The authors declare that the research was conducted in the absence of any commercial or financial relationships that could be construed as a potential conflict of interest.

