# Supplementary material for: Event- and Time-Driven Techniques Using Parallel CPU-GPU Co-processing for Spiking Neural Networks
Source: Front Neuroinform. 2017 Feb 7;11:7. doi: 10.3389/fninf.2017.00007 (PMC5293783; doi:10.3389/fninf.2017.00007)
Supplement: Supplementary file 1 [file DataSheet1.pdf]

APPENDIX A

LIF, AdEx and HH models are here described in full detail. They have been configured in such a way that their I-F curves are similar and generate similar activity levels at the neural system. Thus, we can properly compare their results over our four tests.

Leaky integrated-and-fire model (LIF)

The neural state is defined by the membrane potential (V), which is expressed by (Eq.1):

$$C\frac{dV}{dt} = -g_L(V - E_L) + I \quad (1)$$

where C denotes the membrane capacitance, g_L the conductance responsible for the passive decay term towards the resting potential, E_L the resting potential and I the synaptic interaction (defined in section d). LIF model (Gerstner and Kistler 2002) has a firing threshold V_T. Once V reaches this threshold, an output spikes is generated and the membrane potential is reset to E_L. The membrane potential remains being E_L for the whole refractory period T_{ref} (all these parameters are included in Table 7).

Adaptive exponential integrated-and-fire model (AdEx)

The neural state is defined by the membrane potential (V) and the adaptation variable (w), which are expressed by (Eq. 2):

$$\begin{aligned} C\frac{dV}{dt} &= -g_L(V - E_L) + g_L\Delta_T e^{(\frac{V-V_T}{\Delta_T})} + I - w \\ \tau_w \frac{dw}{dt} &= a(V - E_L) - w \end{aligned} \quad (2)$$

where C denotes the membrane capacitance, g_L the conductance responsible for the passive decay term towards the resting potential, E_L the resting potential and Δ_T the threshold slope factor. V_T is the effective threshold potential, I the synaptic interaction (defined in section d), τ_w the adaptation time constant and a the sub threshold adaptation conductance. AdEx model (Brette and Gerstner 2005) has a reset threshold at zero mV. When V exceeds this threshold, an output spikes is generated and the state variables are set to V=V_r and w=w+b. V_r denotes the reset potential and b the spike triggered adaptation mechanism (all this parameters are included in Table 7).

Hodgkin-Huxley model (HH)

The neural state is defined by the membrane potential (V) and three dimensionless gating variables (m), (h) and (n) that take values between 0 and 1. These variables are associated with the sodium channel activation, the sodium channel inactivation and the potassium channel activation respectively (Eq. 3):

$$\begin{aligned} C\frac{dV}{dt} &= -g_L(V - E_L) - \bar{g}_{Na}m^3h(V - E_{Na}) - \bar{g}_K n^4(V - E_K) + I \\ \frac{dm}{dt} &= \alpha_m(V)(1 - m) - \beta_m(V)m \\ \frac{dh}{dt} &= \alpha_h(V)(1 - h) - \beta_h(V)h \\ \frac{dn}{dt} &= \alpha_n(V)(1 - n) - \beta_n(V)n \end{aligned} \quad (3)$$

where C denotes the membrane capacitance, g_L the conductance responsible for the passive decay term towards the resting potential and E_L the resting potential. $\bar{g}_{NA}$ and $\bar{g}_K$ are the maximum value of the sodium and potassium conductances and I the synaptic interaction (defined in section d) (all these parameters are included in Table 7). HH model equations (Hodgkin and Huxley 1952) do not inherently implement a firing threshold. Conversely, EDLUT needs an eventual threshold for the membrane potential as to predict a spike generation. Setting a functional firing threshold (-30 mV) allows us to simulate HH models using EDLUT computational kernel. α_i and β_i are rate constants for the ith ion channel which is only voltage-dependent (Eq. 4). α_i and β_i are pre-compiled and stored in a look-up table for time-driven neural models running in CPUs. Pre-compiling α_i and β_i parameters in look-up tables is not convenient for GPU neural models since the RAM memory access is neither coalescent nor constant for all neurons at the same time.

$$\begin{aligned} \alpha_m &= 0{,}32\frac{13 - V + V_T}{e^{\frac{(13-V-V_T)}{4}} - 1}; & \beta_m &= 0{,}28\frac{V - V_T - 40}{e^{\frac{(V-V_T-40)}{5}} - 1} \\ \alpha_h &= 0{,}128\ e^{\frac{(17-V+V_T)}{18}}; & \beta_h &= \frac{4}{1 + e^{\frac{(40-V+V_T)}{5}}} \\ \alpha_n &= 0{,}032\frac{15 - V + V_T}{e^{\frac{15-V+V_T}{5}} - 1}; & \beta_n &= 0{,}5e^{\frac{10-V+V_T}{40}} \end{aligned} \quad (4)$$

where V_T adjusts the threshold potential.

Synapse properties

All these models hold the same synaptic interaction mechanism based on excitatory and inhibitory conductances. Equation 5 defines the input current injected to each neuron and the exponential decay functions for both conductances:

$$\begin{aligned} I &= -g_{AMPA}(t)(V - E_{AMPA}) - g_{GABA}(t)(V - E_{GABA}) \\ g_{AMPA}(t_1) &= g_{AMPA}(t_0)e^{\frac{t_0 - t_1}{\tau_{AMPA}}} \\ g_{GABA}(t_1) &= g_{GABA}(t_0)e^{\frac{t_0 - t_1}{\tau_{GABA}}} \end{aligned} \quad (5)$$

where V represents the membrane potential and E_{AMPA} and E_{GABA} the reversal potential of the excitatory and inhibitory synaptic conductances. g_{AMPA} and g_{GABA} stands for the

excitatory and inhibitory conductances that integrate all the contributions received through individual synapses. Both conductances are defined as exponential decay function where τ_{AMPA} and τ_{GABA} represent the exponential decay time constants, t_0 the last ending time and t_1 the current time (all these parameters are included in Table VII).

Whilst the LIF model includes the refractory period, the HH model includes the depolarization and hyperpolarization periods. The maximum operating frequencies for these models are constrained by these time periods. Conversely, the maximum operating frequency for an AdEx model depends on several parameters. Setting the AdEx excitatory conductance to a maximum of 30 nS, limits its maximum operating frequency without modifying the AdEx internal dynamics.

APPENDIX B

One arising question derived from the simulation methods here proposed is the possibility of combining event-driven and time-driven integration methods in the same simulation. The EDLUT simulator allows us to not only evaluate the accuracy of these simulation methods individually but also the accuracy obtained when they are combined. This appendix compares the simulation accuracy results obtained when combining event- and time-driven methods with the results obtained when event-driven and time-driven methods are used separately.

To perform this comparison, a “control neural network” was first defined to be used as an initial reference. This control neural network was the original benchmark configuration (see section 2.4.1 Simulation parameter analysis) in which the second and third neural layers were computed by either event-driven methods only or time-driven methods only. We compared the “control benchmark configuration” with two “combined” neural configurations for the three neural models described (LIF, AdEx and HH):

- A first configuration in which the second neural layer was computed by event-driven methods and the third neural layer was computed by time-driven methods.
- A second configuration in which the second neural layer was computed by time-driven methods and the third neural layer was computed by event-driven methods.

For the sake of simplicity, only the direct method was taken into account amongst the event-driven integration methods. The combined and synchronous event-driven methods presented quite similar behaviors in terms of accuracy. Each neural model used two different look-up table sizes taken from Figure 4: LIF: 29 MB worst and 249 MB best case scenario, AdEx: 81 MB worst and 712 MB best case scenario and HH: 303 MB worst and 1195 MB best case scenario.

Similarly, only the fixed-step and bi-fixed-step integration methods in CPU were taken into account amongst time-driven integration methods. The GPU time-driven methods presented quite similar behaviors in terms of accuracy. Each neural model was simulated using different integration step sizes [0 - 1 ms]. The local step size for bi-fixed-step integration methods was 0.25 ms for both LIF and AdEx models, and 1/15 ms for HH model.

The uncombined simulation methods define a region (Fig. 1, left column) delimited by the worst and the best accuracy results obtained when simulating our “control neural network”. As expected, the results obtained for the combined simulation methods are mostly included in this region. The combined simulations present comparable accuracy results with respect to the uncombined event-driven or time-driven simulation methods. As shown in Figure 1, when the integration step size tends to zero, the accuracy obtained with time-driven methods improves, whilst event-driven methods provide worse accuracy. Combined methods obtain accuracy results right in between these two extremes; not as good as time-driven methods but not as bad as event-driven methods. Likewise, when the simulation step size becomes larger (1ms), the event-driven method accuracy remains better than the time-driven method accuracy. The combined methods, again, obtain accuracy results approximately in between these two methods.

Figure 1 also reveals a fact that ought to be taken into consideration when combining event- and time-driven methods. The sequence in combining event- and time-driven methods for simulating consecutive neural layers matters. The commutative property cannot be applied. The accuracy obtained with the event- and time-driven network configuration and the time- and event-driven network configuration differs. The event- and time-driven combination always presents higher van Rossum distances (worse accuracy) than the time- and event-driven combination. This is caused by two main factors:

- Worse accuracy is usually obtained by event-driven methods compared to time-driven methods in equal conditions. A second neural layer using event-driven methods delivers larger accuracy errors into the third neural layer. These accuracy errors are then propagated and increased in the third neural layer.

- Event-driven methods, in the second neural layer, generate spikes asynchronously, that is, with a high time resolution. However, time-driven methods, in the third neural layer, process all the input spikes synchronously at each global integration step time. That is, all the inputs spikes are computed in time slots of multiple of the global integration step. The time resolution obtained by the second neural layer is diminished by the third neural layer.

The benchmark used here, due to its particular features (i.e. connectivity ratios, firing activity, etc.), does not offer the best deal for event- and time-driven combined methods in terms of performance. On the contrary, as demonstrated in (Naveros et al. 2015), some cerebellar-based benchmarks are able to use event- and time-driven combined methods to improve computational performance without compromising accuracy. The optimal approach is to adopt an event-driven scheme for sparse activity neurons and a time-driven scheme for intensive activity neurons. As argued throughout this paper, choosing time-driven only, even-driven only or event- and time-driven combined methods is always a tradeoff between accuracy, computational performance and the peculiarities of the neural network to be computed.

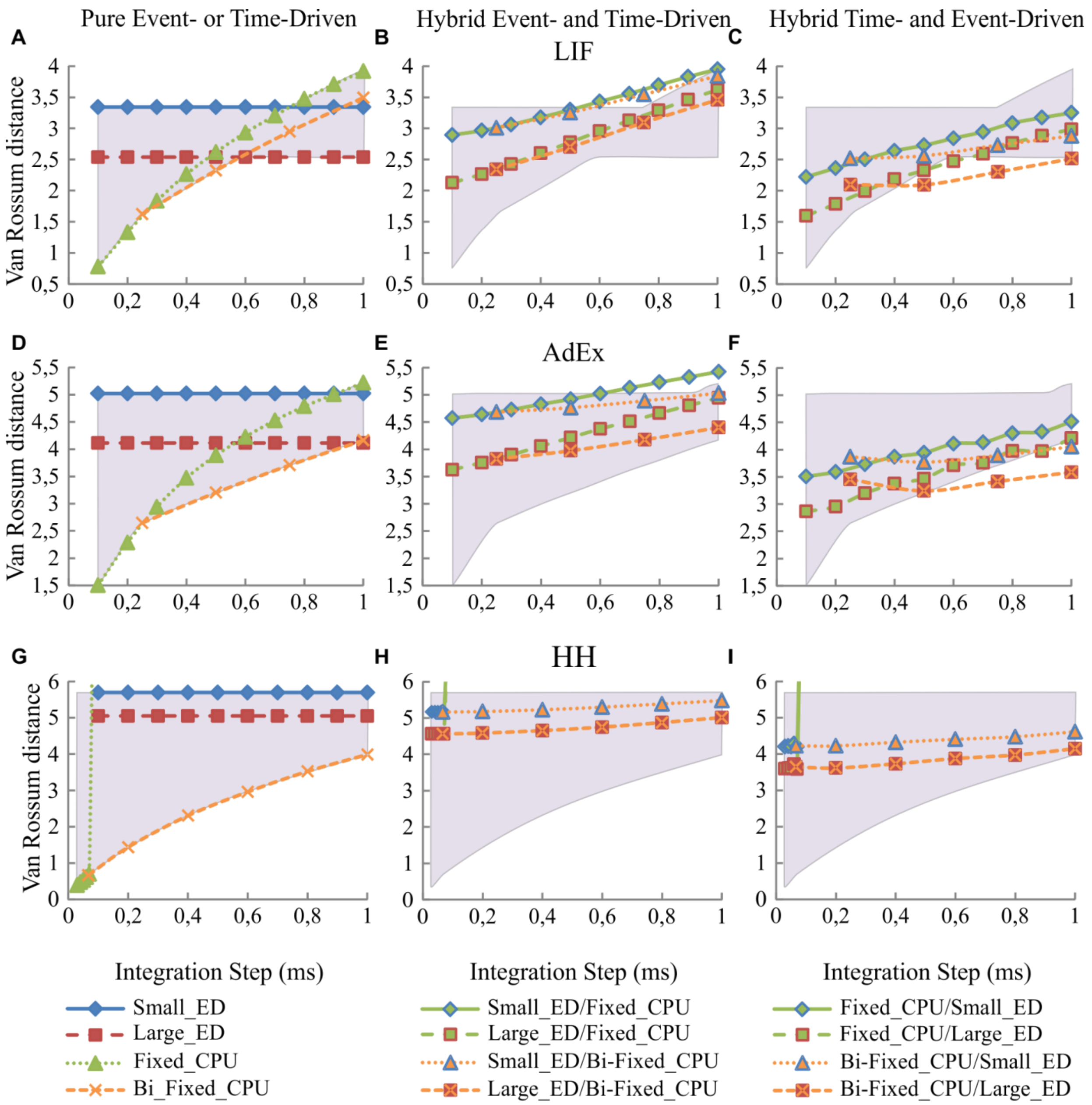

FIGURE 1 | Simulation accuracy of uncombined event- and time-driven methods vs combined event- and time-driven methods for LIF, AdEx and HH neural models. A 1-s simulation of the neural networks defined in **Table 3** is shown. Five different input spike patterns of 5 Hz that generate mean firing rate activities of 10 Hz in the third neural layer are used. The left-hand column (**A,D,** and **G**) of the panel shows the simulation accuracy when either event-driven methods only or time-driven methods only are used in the second and third neural layer. The central column (**B,E,** and **H**) shows the accuracy results obtained when combining event-driven methods for the second neural layer and time-driven methods for the third neural layer. The right-hand column (**C,F,** and **I**) shows the accuracy results obtained when combining time-driven methods for the second neural layer and event-driven methods for the third neural layer. Worst and best accuracy results obtained by the uncombined simulation methods (left column) define the boundaries of an accuracy area (grey shaded area). This area is compared with the accuracy results obtained with the combined simulation methods (central and right columns). The accuracy results obtained by these combined methods are expected to define a boundary area mostly included within the area defined by the uncombined simulation methods. The direct event-driven methods use two look-up table sizes per each neural model (LIF: 29 and 249 MB; AdEx: 81 and 712 MB; HH: 303 and 1,195 MB). The fixed and bi-fixed-step integration methods in CPU evaluate several integration step sizes for each neural model. The global integration step size for fixed and bi-fixed-step integration methods is plotted over x-axis. The standard deviation of the simulation accuracy obtained is negligible; we only represent the mean values.
